# eQTL mapping of the 12S globulin cruciferin gene *PGCRURSE5* as a novel candidate associated with starch content in potato tubers

**DOI:** 10.1038/s41598-020-74285-5

**Published:** 2020-10-13

**Authors:** Dorota Sołtys-Kalina, Katarzyna Szajko, Emil Stefańczyk, Paulina Smyda-Dajmund, Jadwiga Śliwka, Waldemar Marczewski

**Affiliations:** Plant Breeding and Acclimatization Institute-National Research Institute, Platanowa 19, 05-831 Młochów, Poland

**Keywords:** Genetics, Genomics, Transcriptomics

## Abstract

Tuber starch content (TSC) is a very important trait in potato *(Solanum tuberosum* L.). This study is the first to use expression quantitative trait loci (eQTL) mapping of transcript-derived markers for TSC in potato. Thirty-four differentially expressed genes were selected by comparing the RNA-seq data of contrasting bulked segregants. For the 11 candidate genes, we determined their relative expression levels across the segregating diploid potato population using RT-qPCR. We detected 36 eQTL as candidate genes distributed on all twelve potato chromosomes, and nine of them overlapped with QTL for TSC. Peaks for two eQTL, eAGPaseS-a and ePGRCRURSE5, were close to the corresponding loci of the large subunit of ADP-glucose pyrophosphorylase (*AGPaseS-a*) and the 12S globulin cruciferin gene (*PGCRURSE5*), respectively. The eQTL peaks for *AGPaseS-a* and *PGRCRURSE5* explained 41.0 and 28.3% of the phenotypic variation at the transcript level. We showed the association of the DNA markers for *AGPaseS-a* and *PGRCRURSE5* with QTL for TSC, and significant correlation between the expression level of *PGRCRURSE5* and TSC. We did not observe a significant correlation between the expression level of *AGPaseS-a* and TSC. We concluded that the cruciferin gene *PGRCRURSE5* is a novel candidate involved in the regulation of starch content in potato tubers.

## Introduction

Most agronomic traits of crop plants are affected by multiple loci, the environment and their interactions. Quantitative trait loci (QTL) mapping is a routine approach for studying the genetic architecture of complex traits. It yields information and the approximate genomic positions of the factors controlling a quantitative trait, but it does not identify its molecular basis^1^. Combining QTL analysis with genome-wide expression profiling has been termed expression QTL (eQTL) mapping or genetic genomics and creates great opportunities for dissecting quantitative traits^2^. eQTL data can be used to examine genome-wide gene expression levels and find candidate genes for a trait of interest. eQTL are empirically divided into two classes: *cis* and *trans*. A *cis*-eQTL represent a polymorphism physically located near the gene itself, and *trans*-eQTL reside at locations distant from the genes, frequently on different chromosomes^3^. The identification of genes underlying a trait can be more effective when the bulked sample analysis (BSA) method is applied. In the BSA protocol, plants with contrasting phenotype from a segregating population are pooled and then commonly screened to identify specific markers^4,5^. In potato (*Solanum tuberosum* L.), RNA pools that consist of genotypes based on contrasting phenotypic or marker data were used to select candidate genes for tuber flesh colour and cooking type^6^.

Potato is one of the most important crop plants in the world. Among numerous characteristics that are subject to selection in potato breeding, tuber starch content (TSC) is one of the most agronomically important. The starch content of potato tubers ranges from 10 to 25% of the tuber fresh weight^7^, and starch biosynthesis and breakdown in potato tubers are fairly well characterized metabolic networks^8,9^. Linkage mapping of QTL for specific gravity or TSC has been performed in numerous experimental populations of diploid potatoes^10–12^ in addition to association studies^8,13^.

Several enzymes involved in starch metabolism have been identified and characterized at the biochemical and molecular levels^14,15^. ADP-glucose pyrophosphorylase (AGPase) is the key enzyme in the regulation of starch metabolism content and quality^7,16^. All higher plant AGPases, including potato, are heterotetramers composed of two large and two small subunits^17^. For the large subunit AGPaseS, three loci were identified on potato chromosomes I, IV and VIII and for the small subunit AGPaseB, two loci on chromosomes VII and XII^1,8^. The locus *AGPaseS-a* on chromosome I co-localizes with QTL for tuber starch and/or sugar content^18,19^. In our previous paper, we described 12 QTL for starch content located on seven potato chromosomes in the diploid potato population 12-3. The gene encoding *AGPaseS-a* was localized within the most important QTL on chromosome I that accounted for 15.2% of the variance in tuber starch content^12^.

Recent advances in ‘omics’ technologies have made great progress in phenotypic variations and genotypic diversity for complex traits in plant sciences. The application of RNA-sequencing (RNA-seq) technologies has changed transcriptome analyses and gene expression studies^20^. High-throughput RNA-seq technology was used to identify eQTL associated with diverse biological processes in tomato^21^ and eQTL related to quantitative trait variation in maize^22^ and to identify gene networks involved in *Verticillium dahliae* disease resistance in potato^23^.

Here, we used a combination of BSA, comprehensive transcriptome analysis of differentially expressed genes (DEGs) and QTL/eQTL mapping to confirm possible candidates involved in the regulation of the starch content in potato tubers.

## Materials and methods

### Plant material

The plant material consisted of the potato diploid population 12-3 (F1 progeny, N = 175) from a cross of the seed parent DG 00-683 and the pollen parent DG 08-28/13. In the F1 progeny, the TSC segregated and was estimated from the ratio of tuber weight in air (g) to that in water (g) as described by Lunden^24^. The mean TSC values (percent fresh weight), evaluated in years 2012–2014, for DG 00-683 and DG 08-28/13 were 20.8% (± 3.5) and 11.8% (± 0.1), respectively^12^. In our previous study, we used population 12-3 for DArT map construction and QTL analysis of the TSC and sucrose content in potato leaves^12^. The F1 individuals were grown in three replications in a random pattern and scored for TSC directly after being harvested. Data set of TSC is presented in Supplementary Table S1. Samples (5 g) of tubers harvested in 2014 were collected in three replications per genotype, immediately frozen in liquid nitrogen and stored at − 80 °C.

### Isolation of total RNA

Total RNA was isolated according to the protocol of Chomczyński and Sacchi^25^ using TRIZOL reagent. Briefly, frozen tubers were ground in liquid nitrogen, and 1 g of ground tissues was taken prior to the addition of 4 ml of TRIZOL reagent. After incubation at room temperature and centrifugation, the supernatants were transferred to fresh tubes. The extraction was performed twice in 3 ml of chloroform. The RNA was precipitated 15 min after the addition of 0.6 ml of salt solution (0.8 M sodium citrate and 1.2 M sodium chloride) and 0.6 ml of isopropanol. The RNA concentration and quality were determined using a biophotometer (Eppendorf) at 260 nm, 280 nm and 230 nm. The RNA was used for reverse transcription polymerase chain reaction (RT-PCR) and quantitative real-time PCR (RT-qPCR) experiments.

### Construction of bulk RNA and Illumina sequencing

For the RNA-seq study, the quality and quantity of the total RNA were established using Bioanalyzer 2100 (Agilent). Four bulk RNA samples were constructed, each with two biological replicates. For each RNA bulk sample, equal amounts of total RNA (1 µg) from the tubers of six plants were pooled together. Bulks H1 and H2 consisted of high TSC genotypes, ranging from 19.0 to 23.4%; bulks L1 and L2 were made of low TSC genotypes, ranging from 12.5 to 15.0%. Plants in bulks H1 and L1 strongly expressed *AGPaseS-a*, whereas those in bulks H2 and L2 exhibited low levels; *AGPaseS*-*a* expression was determined as described in^12^. The mRNA was isolated using the NEBNext Poly(A) mRNA magnetic Isolation Module (New England Biolabs, E7490), and cDNA libraries were prepared using the NEBNext Ultra Direction RNA Library Kit for Illumina (New England Biolabs, E7420S). The established cDNA libraries were sequenced on the Illumina HiSeq 4000 sequencing platform (Illumina Inc., San Diego, CA, USA) to generate 100-bp paired-end reads (PE100). RNA-seq reads were conducted by Genomed S.A. (Warsaw, Poland). Quality control (QC) was done using Trimmotatic software (FASTQ-Illumina Phred + 33) for the raw data, which were trimmed by removing all empty and low-quality reads (Q < 30 and length < 50 bp), as well as all adaptor sequences, in order to obtain clean reads. The QC data are shown in Supplementary Table S2. Then, the index of the reference genome (https://www.ncbi.nlm.nih.gov/assembly/GCF_000226075.1) was built using Bowtie v2.1.0, and paired-end clean reads obtained for bulks H and L were aligned to the reference genome using TopHat v2.0.9 (Broad Institute, Boston, MA). Next, HTSeq v0.5.3 was used to count the number of reads mapped to each gene. The DEGs were identified by DESseq package. A comparison of DEGs from bulks H and L is shown in Supplementary Table S3. A scheme of the methodology used in this study is shown in Supplementary Fig. S1.

### Selection of differentially expressed genes

DEGs were identified based on the RNA-seq data by comparing H1 vs. L1 and H2 vs*.* L2, H1 and H2 were control samples. Data from these comparison represents up-regulated and down-regulated genes in bulks L1 and L2. A false discovery rate (FDR) of 0.05 and absolute values of log2 ratios ≥ 1.5 and ≤ − 1.5 for up- and downregulated genes were used as the threshold for determining the significance of gene expression differences. To confirm RNA-seq data and develop the transcript-derived markers, semi-quantitative RT-PCR assays were performed on bulked samples. Reverse transcription was performed using the PrimeScript Master Mix (TaKaRa, no. #RR036A) cDNA synthesis kit, and 2 µg of total RNA was used for each reaction. Semi-quantitative RT-PCR was performed using DreamTaq DNA polymerase (Thermo Fisher Scientific, No. EP0703) and products were visualized in 2% agarose gel with ethidinum bromide. The primer sequences and PCR condition applied in semi-quantitative RT-PCR are presented in Supplementary Table S4.

### Construction of the genetic map with transcript-derived markers

Standard PCR markers were developed using the selected candidate gene sequences. The primer sequences and PCR parameters for amplification of cleaved amplified polymorphic sequence (CAPS) and one sequence characterized amplified region (SCAR) markers are described in Supplementary Table S5. JoinMap 4 software^26^ was used for mapping on the previously constructed DArT map, as described in^12^.

### eQTL mapping

The expression of the ten selected candidate genes and *AGPaseS-a* was examined in the F1 progeny of the 12-3 population by RT-qPCR. SYBR Green PCR Master Mix (Roche, Switzerland) and 96-well plates with a LightCycler 480 II system (Roche, Switzerland) were used. The RT-qPCR was performed as previously described in Śliwka et al*.*^12^. The 1 μl of cDNA corresponding to 50 ng of total RNA was taken for analysis of each sample. Potato α-tubulin was used as the reference gene. Thermal cycling conditions were: 4 min denaturation at 95 °C followed by 55 cycles of 10 s at 90 °C, 20 s at temperature for primer annealing, and 30 s at 72 °C. To confirm amplification of gene-specific products, PCR product melting point was determined in the range of 65–97 °C. The primer sequences and RT-qPCR parameters are shown in Supplementary Table S6. Four technical replicates of the parents and F1 progeny of population 12-3 were performed. Relative expression levels were calculated in Microsoft Excel 2010. *T* tests for ΔΔ*C*t cycle threshold values^27^ and calculation of standard errors of the mean (SE) were performed with Statistica software (Stat Soft Inc.). For eQTL mapping, MapQTL 6 software was utilized^28^, with internal mapping and a logarithm of odds (LOD) ≥ 3.0 as the threshold of significance. The Pearson correlation coefficient (*r*) and the probability value (*p*) were used to display correlations and the significance of differences in expression levels between the candidate genes and TSC. A probability value of *p* < 0.05 was considered to indicate statistical significance.

Additionally, the expression profiles of *PGRCRURSE5* and *AGPaseS-a* were examined in tubers of the parental clones DG 00-683 and DG 08-28/13 at three developmental stages: stage I, the beginning of tuber formation (1 cm diameter tubers); stage II, tuber building, tuber approximately 2 cm diameter; and stage III- tuber maturity. The experiment was carried out in the same way as that for candidate gene expression in the F1 progeny of the 12-3 population.

### Cloning and sequencing of PGCRURSE5 amplicons

PCR-based amplicons 1587 bp in size for the marker PGCRURSE5 were obtained from both parents and purified using a Clean-Up Kit (A&A Biotechnology, Gdynia, Poland) according to the manufacturer’s protocol. The amplicons were blunted using a Fast DNA End Repair Kit (Thermo Fisher Scientific) and cloned into a blunt pCRScript Amp SK cloning vector (Promega, Madison, Wisconsin, USA). *E. coli* Top10 chemocompetent cells were used for transformation, and colonies with inserts of interest were picked and sequenced bidirectionally. Sequencing reactions were performed using the BigDye Terminator v3.1 kit (Life Technologies Polska Ltd., Warsaw, Poland), and products were resolved on an ABI3730XL genetic analyser at the Laboratory of DNA Sequencing and Oligonucleotide Synthesis (Institute of Biochemistry and Biophysics, Polish Academy of Sciences, Warsaw, Poland).

## Results

### Sequencing data, differential expression gene analysis and genetic mapping

A total of eight bulk samples of tuber RNA were analysed by Illumina sequencing. Altogether, over 497 million reads were generated, with the number of RNA-seq reads per library ranging from 29 to 41 million after filtering impurities (Supplementary Table S2). All raw and processed data have been deposited in the GEO database (GSE153031) under the link: https://www.ncbi.nlm.nih.gov/geo/query/acc.cgi?acc=GSE153031.

Two pairwise comparisons were performed. When H1 was compared to L1, ten genes were upregulated and 13 genes were downregulated significantly (*P* value < 0.05 and fold change ≥ 1.5 or ≤  − 1.5). The corresponding values for H2 *vs.* L2 data were 4 and 7. For only 15 candidate genes, differences in transcript-intensities between the bulks were observed using semi-quantitative RT-PCR (data not shown). The selected genes are listed in Table 1. DNA markers were developed for eight gene sequences from the comparison of H1 vs. L1 and for the *SWEET12-like* gene from the comparison of H2 vs. L2. The CAPS and SCAR markers were scored in population 12–3 and incorporated into the existing genetic map (Table 2, Supplementary Table S7). The DNA marker AGPaseS-a was mapped in our previous study^12^. Markers for *UnCh865* and *WAT1* were not mapped in population 12-3. The positions of the gene *WAT1* and the uncharacterized gene *UnCh865* were deduced from their positions on the physical map of the reference genome DM1-3 v4.03 and the positions of the closest DArT markers that were common to genetic map 12-3 and the physical reference map (Table 2).

**Table 1:** The list of the DEGs in comparison of the bulks H vs. L, obtained by imposing a hard cut-off (FDR < 0.05; Log2FC ≥ 1.5 or Log2FC ≤ − 1.5).

Locus	Marker	Gene name	log2FC^a^	FDR
**Bulks**^b^				
**H1/L1**				
LOC102601158	PGCRURSE5	Cruciferin PGCRURSE5-like transcript variant X1	2.09	0.0002
LOC102593485	R1B-23	Putative late blight resistance protein homolog R1B-23 transcript variant X2	2.08	0.0046
LOC107057670	WAT1	WAT1-related protein At1g09380-like	1.88	0.0011
LOC102580754	ANR	Anthocyanidin reductase-like	1.86	0.0288
LOC102588225	9-DES	9-Divinyl ether synthase	− 3.32	0.0000
LOC102584283	MLP34	MLP-like protein 34	− 1.90	0.0126
LOC102603835	Unch835	Uncharacterized LOC102603835	− 1.90	0.0250
LOC102596717	IRL	Isoflavone reductase-like protein	− 1.81	0.0020
LOC107059465	Pat3-k1	Probable inactive patatin-3-Kuras 1	− 1.71	0.0023
**H2/L2**				
LOC102591902	SWEET12-like	Bidirectional sugar transporter SWEET12-like	1.54	0.0047
LOC102598865	Unch865	Uncharacterized LOC102598865	1.53	0.0038
LOC107059838	Unch838	Uncharacterized LOC107059838 transcript variant X2	− 1.85	0.0001
LOC102579204	GST	Probable glutathione S-transferase	− 1.58	0.0037
LOC102590434	PI-2	Proteinase inhibitor type-2-like	− 1.55	0.0045
LOC102594285	ECERIFERUM 3-like	Protein ECERIFERUM 3-like transcript variant X2	− 1.53	0.0029

^a^Log2 estimated fold change.

^b^Bulks H1 and H2 were for high tuber starch content genotypes; L1 and L2 were for low tuber starch content genotypes. H1 and L1 indicated high expression of *AGPaseS-a*; H2 and L2 were for its low expression; H1 or H2 were control bulks

**Table 2:** List of DNA markers developed for candidate genes and their effect on their own expression (*cis-*eQTL) and on the starch content (QTL for starch content) in potato tubers of population 12-3.

Marker	Group	Position on genetic map 12-3 (cM)	*cis*-eQTL		QTL for starch content	
			LOD	*R*^*2*^ (%)^a^	LOD	*R*^*2*^ (%)^a^
UnCh835	I	24.4	ns^b^		ns	
PGCRURSE5	I	80.4	11.26	25.9	8.30	18.8
9-DES	I	91.1	ns		8.08	18.4
AGPaseS-a^c^	I	99.6	19.44	40.8	8.13	18.5
IRL	IV	5.9	ns		3.17	7.7
R1B-23	IV	48.0	7.48	18.1	ns	
ANR	IV	70.8	3.10	7.9	ns	
SWEET	V	51.3	nt^f^		ns	
Pat3-k1	VIII	14.6	ns		ns	
UnCh865	VIII	31.8 (pPt-656292)^d^	ns		3.34	8.1
MLP34	IX	7.3	ns		ns	
WAT1	XII	56.1–63.2 (pPt-533837-pPt-650660)^e^	ns		ns	

^a^Percent of the variance explained.

^b^Not significant.

^c^Corresponded to the marker allele AGPaseS-a1334 described by Śliwka et al. ^12^.

^d^Marker Unch865 was not mapped in the population 12-3, the marker pPt-656292 is located in the reference genome DM1-3 v4.03 at chr08:45136238..45135798, closest to the location of the Unch865 (chr08:45297205..45303745).

^e^Marker WAT1 was not mapped in the population 12-3, the interval pPt-533837–pPt-650660 is located in the reference genome DM1-3 v4.03 at chr12:16186839..50172657, which is including the position of WAT1 (chr12:38101094..38105242).

^f^Not tested.

### Genetic positions of the candidate gene markers relative to their eQTL and QTL for TSC

For 11 candidate genes, we determined the relative expression levels in the tubers of all F1 individuals of population 12-3 by RT-qPCR (Supplementary Table S8) and used the results for eQTL analysis. The expression of the *SWEET12-like* gene was not measured in the F1 individuals (Table 2). Four candidate gene markers, PGCRURSE5, AGPaseS-a, R1B-23 and ANR, were mapped within the regions corresponding to the eQTL controlling their expression (*cis*-eQTL). However, only the markers PGCRURSE5 and AGPaseS-a were significantly associated with QTL for TSC and explained 18.8 and 18.5% of the variation in TSC, respectively. The PGCRURSE5 marker, 1587 bp in size, was cloned, sequenced and compared to the database with the BLASTN programme (NCBI database). We found two sequences in the DG 00-683 parent (GenBank accessions MT274591 and MT274592) and one sequence for this marker in the parent DG 08-28/13 (MT274590). This marker from the DG 00-683 parent shared 95% sequence identity with the *Solanum tuberosum* 12S seed storage protein CRD-like (alternative name: Cruciferin D; UniProt accession Q9ZWA9-1, GenBank accession XM_006349369.2). The HpaII recognition site was diagnostic of the *PGCRURSE5* marker allele in parent DG 00-683. In contrast, none of the DG 08-28/13-derived PGCRURSE5 sequences contained the HpaII site. The marker 9-DES, although also mapped on chromosome I, was located outside the eQTL for the *9-DES* region but within the QTL for TSC, and accounted for 18.4% of the variance in this trait. The locus of the gene *UnCh865* was on chromosome VIII within the QTL with a moderate effect on TSC (LOD = 3.34; R^2^ = 8.1%) but not within the eQTL for *UnCh865*. The markers Pat3-k1, MLP34 and WAT1 were located in regions not affecting the expression of their genes or TSC (Table 2).

### eQTL analyses

In our previous study, we performed QTL analysis of TSC using a phenotypic mean dataset (2012–2014) in population 12-3^12^. In the current research, the TSC linkage map was enriched by a set of 9 DNA markers, for which we found polymorphism in parents and F1 individuals (Table 2, Supplementary Table S7). For 11 genes, expression products were obtained in RT-qPCR (Table 3 and Supplementary Fig. S2). The number of eQTL detected for particular candidate genes ranged from one (*AGPaseS*) to seven (*ANR*). We found eQTL located both close to the loci encoding the genes (*cis*-eQTL) and at independent locations (*trans-*eQTL). In total, 36 eQTL were mapped in population 12-3 (Table 3).

**Table 3:** QTL for TSC (mean 2012–2014) and eQTL for the selected candidate genes in potato tubers of population 12-3.

Chromosome	Trait	Peak location (cM)	Marker at peak or markers flanking peak interval	LOD	*R*^*2*^ (%)^a^	QTL/eQTL location (cM)
**I**	TSC	0.0	pPt-536705	4.75	11.3	0.0–4.5
	TSC	28.6	pPt-537757	3.46	8.3	28.6–29.7
	TSC	80.7	pPt-536041	9.35	21.0	38.5–103.2
	eAGPaseS	99.3	pPt-535812	19.61	41.0	70.8–105.9
	ePGCRURSE5	84.3	pPt-471128	12.48	28.3	58.1–102.7
	eR1B-23	26.7	pPt-458558	32.81	58.2	26.7–26.8
	eR1B-23	104.5	pPt-458410	9.50	22.4	103.2–106.0
	e9-DES	27.8	pPt-471648	19.22	42.9	27.7–28.5
	eUnCh865	27.8	pPt-471648	3.17	9.5	27.7–27.8
**II**	TSC	26.4	pPt-540301	4.30	10.3	23.8–28.4
	TSC	54.8	pPt-656727	4.38	10.4	35.0–68.0
	eIRL	62.5	pPt-471130	3.28	8.4	62.5–62.6
	eWAT1	51.4	pPt-470784	7.07	17.2	50.0–52.7
**III**	TSC	68.1	toPt-437014–pPt-538033	3.90	9.3	65.1–71.1
	TSC	130.5	pPt-656987	3.24	7.8	129.4–130.5
	ePGCRURSE5	64.1	toPt-437014	4.33	10.9	62.3–68.1
	eANR	3.0	pPt-456510–toPt-437382	4.75	11.9	1.0–3.2
	eANR	46.0	pPt-539332	6.33	15.5	44.7–46.6
	eANR	61.3	capPt-672649–toPt-437014	10.17	23.5	60.1–64.1
	e9-DES	29.8	pPt536814	4.75	12.9	29.6–29.8
	eMLP34	130.5	pPt-656987	3.70	9.4	124.4–130.5
**IV**	TSC	49.6	pPt-535592	3.16	7.6	49.5–50.2
	eR1B-23	6.1	pPt-458171	5.27	13.1	5.6–7.8
	eR1B-23	10.2	toPt-439845–pPt-459479	15.69	34.1	8.8–14.2
	eR1B-23	40.0	pPt-458273	3.60	9.1	37.5–40.0
	eR1B-23	59.3	PEPCb	10.87	25.1	44.3–95.1
	eANR	70.3	pPt-537828–ANR	3.29	8.4	70.3–70.8
	eWAT1	44.3	pPt-536644–pPt-539002	3.61	9.2	44.3–45.4
**V**	TSC	49.3	pPt-471563	3.55	8.6	46.0–50.7
	eANR	4.7	pPt-539686	19.14	39.9	4.7
	eUnCh865	55.6	pPt-539891	5.07	14.8	40.5–55.6
**VI**	eANR	35.8	pPt-536287	6.46	15.8	34.1–35.8
	eUnCh835	29.0	pPt-538867	5.74	14.2	28.9–29.8
	eIRL	51.2	pPt-539557	12.38	28.1	37.9–51.2
**VII**	eANR	36.8	pPt-536728	34.16	59.7	36.8–58.2
	eIRL	5.0	pPt-653355	3.77	9.6	4.6–7.1
	eWAT1	3.4	pPt-652595	8.81	20.9	0.0–8.1
**VIII**	TSC	39.0	B-amyl	5.74	13.5	26.0–40.6
	TSC	63.8	toPt-436952	3.95	9.5	49.7–63.8
	eMLP34	64.1	pPt-651102	3.79	9.6	63.8–64.1; 40.5
	eUnCh835	40.6	pPt-656606	10.12	23.6	40.3–41.6
**IX**	eMLP34	19.5	pPt-458119	6.31	15.5	12.3–26.4
**X**	TSC	28.8	pPt-534845	6.65	15.4	16.5–46.5
**XI**	TSC	54.1	pPt-471789	5.04	11.9	49.8–55.8
	TSC	66.5	pPt-473204–pPt458659	4.30	10.3	58.1–74.14
	e9-DES	49.7	pPt-652888	7.72	20.2	49.7
	e9-DES	81.4	pPt-535328	15.82	36.9	81.3–81.5
**XII**	TSC	143.5	pPt-656237	5.51	12.9	117.7–143.5
	ePGCRURSE5	11.0	SUS4a–SUS4b	5.49	13.6	0.0–24.7
	eANR	43.7	pPt-657438	12.76	28.8	43.5–50.7
	e9-DES	65.1	pPt-536961	8.61	22.2	63.2–65.7

^a^Percent of the variance explained.

^b^Bulks H1 and H2 were for high tuber starch content genotypes; L1 and L2 were for low tuber starch content genotypes. H1 and L1 indicated high expression of *AGPaseS-a*; H2 and L2 were for its low expression; H1 or H2 were control bulks

### Colocalization of eQTL with QTL for TSC

**Figure 1:**
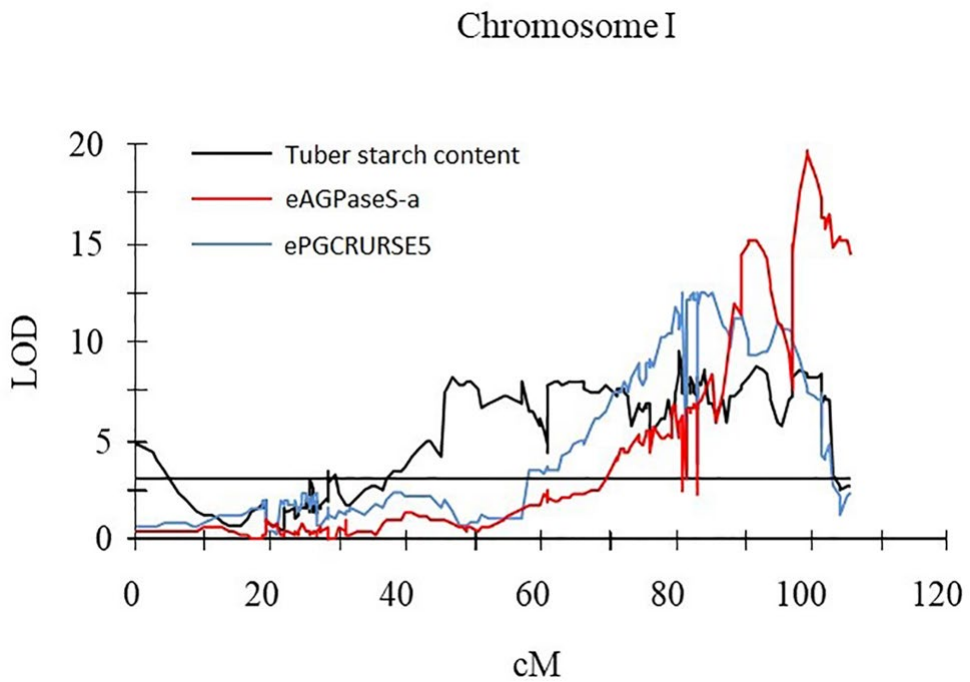
LOD charts of QTL detected by interval mapping for the 3-year mean (2012–2014) tuber starch content and eQTL for *AGPaseS* and *PGCRURSE5* candidate genes in the diploid potato mapping population 12-3. Threshold LOD = 3, marked by a line parallel to the x-axis.

Nine of the 36 eQTL identified for candidate genes overlapped with QTL for TSC (Table 3). On chromosome I, two eQTL, for *AGPaseS-a* and *PGRCRURSE5*, overlapped with the strongest QTL for TSC (Fig. 1). The eQTL peaks for *AGPaseS* and *PGRCRURSE5* were found at 99.3 and 84.3 cM, explaining 41.0 and 28.3% of the variance in the expression of these genes, respectively (Table 3). Both peaks were located near the loci encoding those genes (99.6 and 80.4 cM, Table 2).

On chromosome II, two eQTL were detected within the QTL for TSC 35.0–68.0 cM: eQTL located at 50.0–52.7 cM and explaining 17.2% of the variance in eWAT1 and located at 62.5–62.6 cM and explaining 8.4% of the variance in eIRL. The second eQTL for *PGRCRURSE5*, found on chromosome III, partially overlapped with the QTL for TSC mapped at 65.1–71.1 cM and explained 10.9% of the variance in ePGRCRURSE5. On chromosome III, we also detected the eQTL for *MLP34* located at 124.4–130.5 cM and explained 9.4% of the variance in the expression of MLP34 that partially overlapped with the QTL for TSC at 129.4–130.5 cM. Chromosome IV possessed eQTL for *R1B-23* that covered 44.3–95.1 cM (R^2^ = 25.1%) and overlapped with the QTL for TSC at 49.5–50.2 cM. On chromosome V, we detected a QTL for TSC at 46.0–50.7 cM that explained 8.6% of the variance in this trait. It overlapped with an eQTL for *UnCh865* located at 40.5–55.6 cM that explained 14.8% of the variance observed in the expression of this gene. An eQTL for *UnCh835* at 40.3–41.6 cM on chromosome VIII overlapped slightly with the QTL for TSC at 26.0–40.6 and explained up to 23.6% of the variance in eUnCh835 (Table 3). In addition, we detected 20 eQTL on chromosomes I, III, IV, V, VIII, IX and XII that were outside the QTL for TSC (Table 3). Seven eQTL were identified on chromosomes VI, VII and IX, whereas no QTL for TSC were found on these chromosomes (Table 3). Significant positive correlations were found between the expression levels of a few pairwise combinations of the candidate genes. The eQTL for *PGRCRURSE5*, *MLP34*, *IRL* and *Pat3-k1* exhibited significant positive correlations with TSC (Supplementary Table S9).

### *AGPaseS-a* and *PGRCRURSE5* expression assay during major stages of tuber development

**Figure 2:**
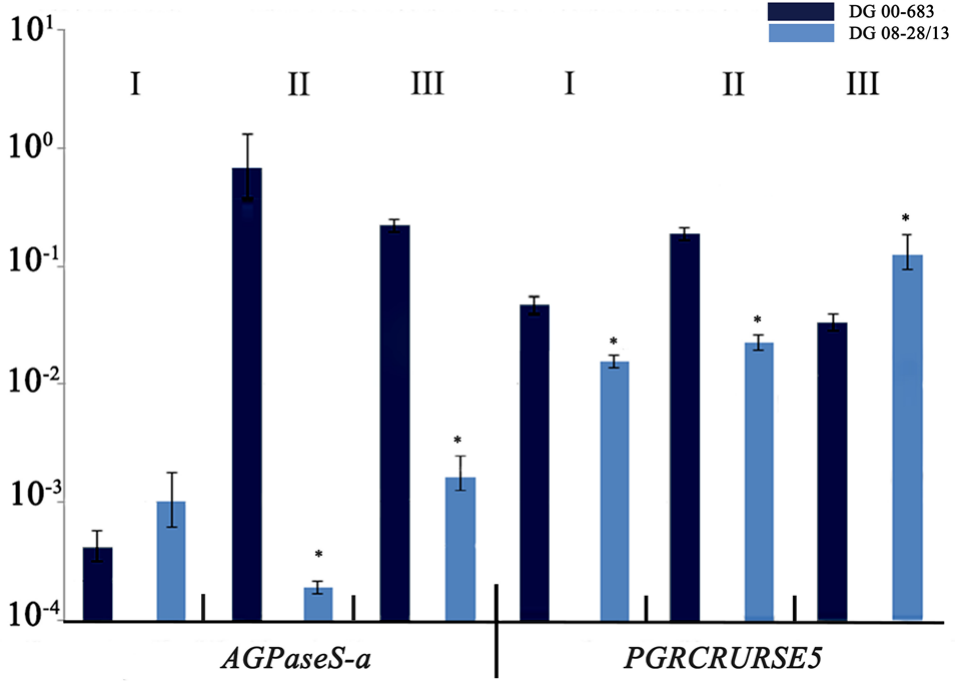
Relative expression levels of the AGPaseS-a and PGRCURSE5 genes in the high-starch parent DG 00-683 and the low-starch parent DG 08-28/13 at three tuber developmental stages: stage I—tuber formation; stage II—tuber building, tubers approximately 2 cm diameter; and stage III—tuber maturity. The levels of relative transcript accumulation are shown on the y-axis (logarithmic scale); values are presented as the means ± SD of three biological replicates. Asterisks indicate significant differences between high- and low-starch parents (Student’s t-test).

The markers PGCRURSE5 and AGPaseS-a were significantly associated with QTL for TSC (Table 2). In addition, eQTL for *AGPaseS-a* and *PGRCRURSE5* overlapped with the strongest QTL for TSC on chromosome I (Fig. 1). Therefore, we evaluated the expression of *AGPaseS-a* and *PGRCRURSE5* during major stages of tuber development. The expression level of *AGPaseS-a* was similar (not significantly different) in both parents at stage I of tuber development and significantly higher in the high-starch parent DG 00-683 than in the other parent at stages II and III. In the case of *PGRCRURSE5*, differential expression was observed between DG 00-683 and DG 08-28/13 at all three stages. *PGRCRURSE5* expression in DG 00-683 was highest at stage II, while in the low-starch parent DG 08-28/13, its transcript level increased during tuber development and was highest at stage III in DG 08-28/13 (Fig. 2).

## Discussion

Starch is the most abundant storage compound in plants. As in other higher plants, starch synthesis in potato is under transcriptional control, circadian and redox control, and phosphorylation regulation^29,30^. The *AGPaseS* loci, in particular, the locus *AGPaseS-a* on chromosome I, colocalized with QTL for TSC, and the data indicated a small effect on this trait in the mapping populations^18,31^. In association studies, the amplicons AGPsS-9a and AGPsS-10a, both derived from the *AGPaseS-a* locus, were correlated either positively or negatively with TSC^32^. Previously, we showed the large QTL region for TSC on potato chromosome I that overlapped with the *AGPaseS-a* locus. This result potentially means that the chromosomal segment also includes other genes that either directly or indirectly affect the starch content. The expression of *AGPaseS-a* was significantly higher in the high-TSC parent DG 00-683 than in the low-TSC parent DG 08-28/13 in the potato population 12-3^12^. The *AGPaseS-a* allele contributed significantly to but was not necessary for a high TSC^12^.

Here, we detected the differences between the parental clones in the expression levels of *AGPaseS-a* in tubers at different growth stages. The highest expression was detected in the tuber building stage, when high AGPase activity is required, as the tuber is a sink organ accumulating large amounts of starch^33^. Our results confirmed that AGPase activity remained high even when starch synthesis was inhibited in potato tubers detached from the mother plant^34^. In the current sequencing experiments, *AGPaseS-a* was difficult to analyse by RNA-seq, which was potentially due to the small transcript size restricted by the constructed RNA libraries and/or sequence overlap with other transcripts^35,36^.

We mapped the cruciferin (12S globulin) gene *PGCRURSE5* to chromosome I and demonstrated that it also had a significant effect on TSC. In *Arabidopsis thaliana* and other crucifers, cruciferin is a main seed storage protein. Seed storage proteins serve as a source of nitrogen and amino acids that are necessary for germination and plant growth^37,38^. In potato tubers, the inhibition of starch synthesis was accompanied by a massive reduction in the expression of storage proteins, suggesting that the expression of storage protein genes is involved in starch metabolism in potato tubers^39^. Our study revealed higher expression of *PGCRURSE5* in the high-starch parent DG 00-683 than in the low-starch parent DG 08-28/13 during the tuber formation and building stage, and we therefore concluded that the cruciferin protein can affect starch metabolism. Among the 9 eQTL identified for the selected candidate genes that overlapped with QTL for TSC, the peaks for two eQTL, *AGPaseS-a* and *PGRCRURSE5*, were close to the loci encoding those genes. In the case of ePGRCRURSE5, the presence of a *trans-*eQTL on chromosomes III and XII showed that *PGCRURSE5* expression is influenced by *trans-*acting factors.

The *cis*-eQTL are likely mediated by polymorphisms within the corresponding genes, including the promoter regions, or by mRNA stability^40^. The overlap of QTL and eQTL may indicate a strong association between the genetic variation in the phenotypic trait and the gene transcript level^41^. The genes for *AGPaseS-a* and *PGRCRURSE5* accounted for 18.5 and 18.8% of the variance in TSC, respectively. The eQTL peaks for *AGPaseS-a* and *PGRCRURSE5* explained 41.0 and 28.3% of the phenotypic variance at the transcript level, respectively. The high association values between eQTL peaks and the explained expression variations could account for genetic sources of variation associated with dominance and epistasis as well as for non-genetic influences, such as developmental and environmental factors^42^. Colocalization of *cis-*eQTL and QTL seems to be more informative than that of *trans*-eQTL. *Trans*-eQTL are interpreted as evidence for *trans*-acting regulatory proteins such as transcription factors and other signalling proteins or small RNAs that may control the expression of a number of genes elsewhere in the genome^43^.

The role of AGPase as the first rate-limiting enzyme in starch biosynthetic pathways is well known^8,44,45^. We showed the association of the DNA marker for *AGPaseS-a* with QTL for TSC as well as the relationship between this marker and gene transcription. However, we did not observe a significant correlation between the expression level of *AGPaseS-a* and TSC. The abundance of mRNA transcripts only partially correlates with protein abundances, and these relationships are complex^46,47^. Therefore, in the case of the enzyme AGPase, their subunit structure and transcriptional regulation can affect the net activity of this enzyme complex.

Our study demonstrates the association between the marker PGCRURSE5 and QTL for total starch content, the relationship between this marker and the eQTL for *PGRCRURSE5*, and significant correlation between *PGCRURSE5* expression and starch content in potato tubers. Recently, Sueng et al*.*^48^ has shown that non-enzymatic protein, termed Protein Targeting to Starch (PTST), is involved in starch synthesis in Arabidopsis. Our results identified the gene cruciferin as a novel candidate involved in the regulation of starch metabolism in potato tubers. It suggests that cruciferin may be a novel PTST protein in potato tubers.

## Supplementary information

Supplementary Information 1.

Supplementary Information 2.

Supplementary Information 3.
