# Supplementary material for: eQTL mapping of the 12S globulin cruciferin gene PGCRURSE5 as a novel candidate associated with starch content in potato tubers
Source: Sci Rep. 2020 Oct 13;10:17168. doi: 10.1038/s41598-020-74285-5 (PMC7553954; doi:10.1038/s41598-020-74285-5)

eQTL mapping of the 12S globulin cruciferin gene *PGCRURSE5* as a novel candidate associated with starch content in potato tubers.

Dorota Sołtys-Kalina*, Katarzyna Szajko, Emil Stefańczyk, Paulina Smyda-Dajmund, Jadwiga Śliwka, Waldemar Marczewski*

Plant Breeding and Acclimatization Institute – National Research Institute, Młochów, Platanowa 19, 05-831 Młochów, Poland

*Corresponding Authors: d.soltys@ihar.edu.pl; w.marczewski@ihar.edu.pl

Supplementary Figure S1. The scheme of the methodology used in this study

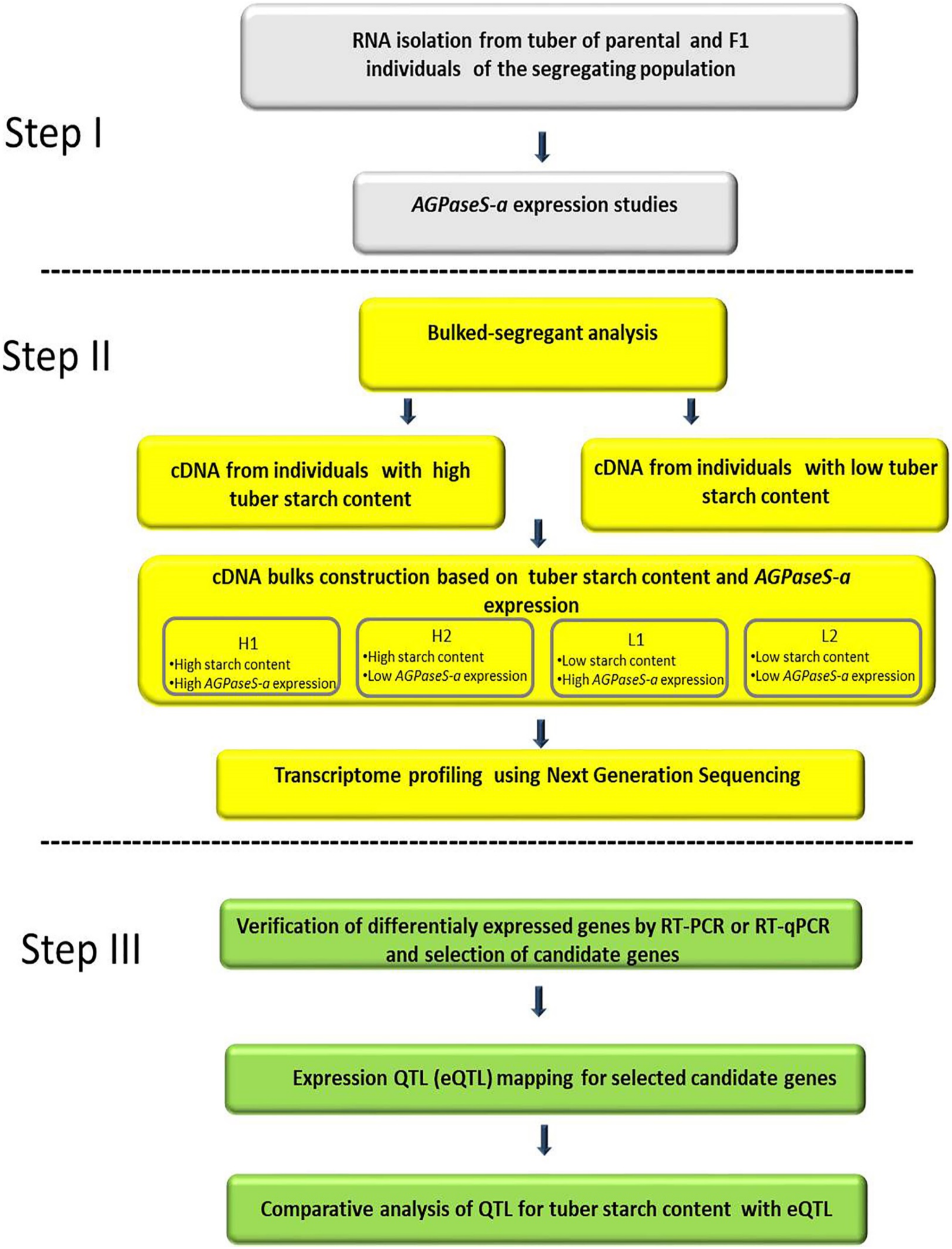

Supplementary Figure S2. QTL for tuber starch content and eQTL for 11 candidate genes. Y-axis: LOD, X-axis: genetic distance in cM. The scales are adjusted to the trait values and differ between traits and chromosomes. The chromosomes (groups) are marked above the charts

Tuber starch content

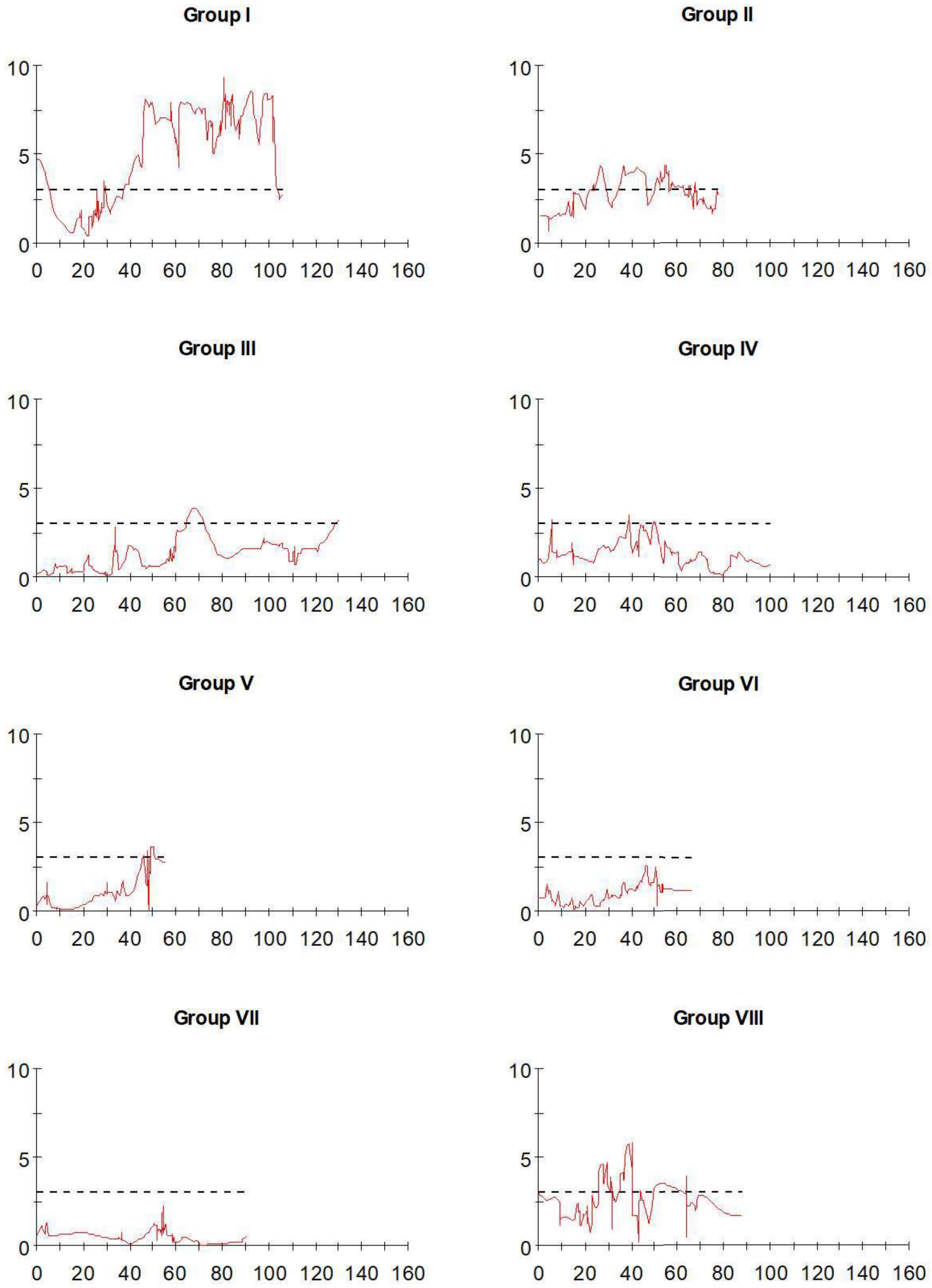

Tuber starch content -continued

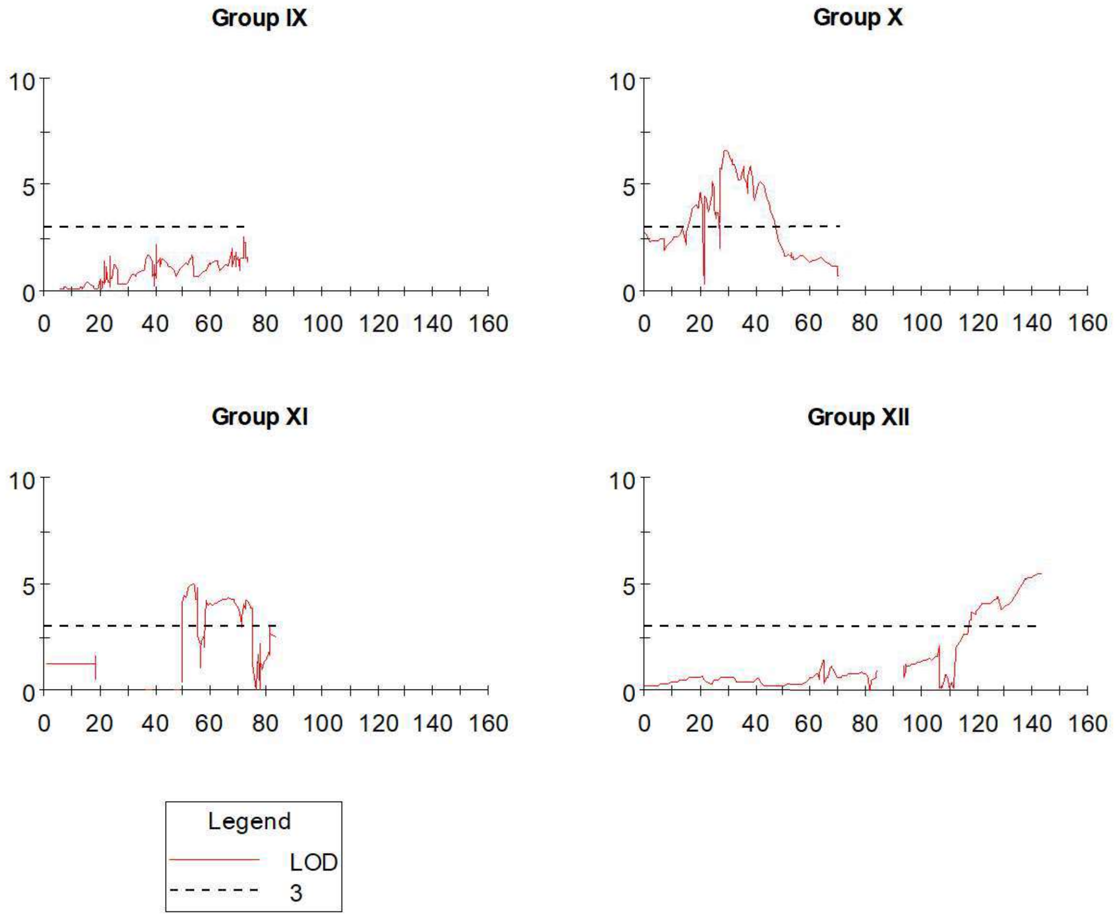

eAGPaseS-a

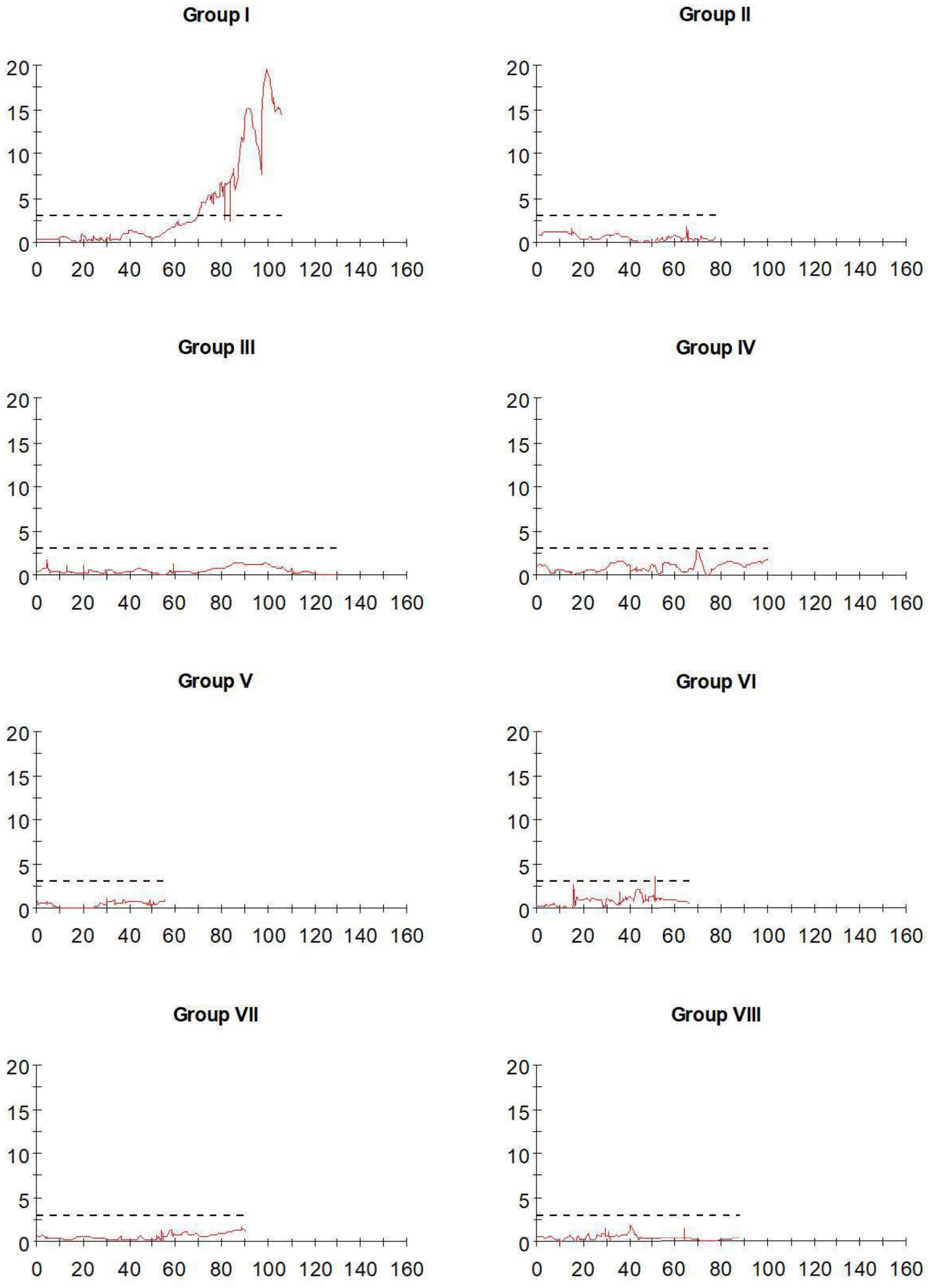
Group I
Group II
Group III
Group IV
Group V
Group VI
Group VII
Group VIII
0 5 10 15 20
0 20 40 60 80 100 120 140 160

eAGPaseS-a - continued

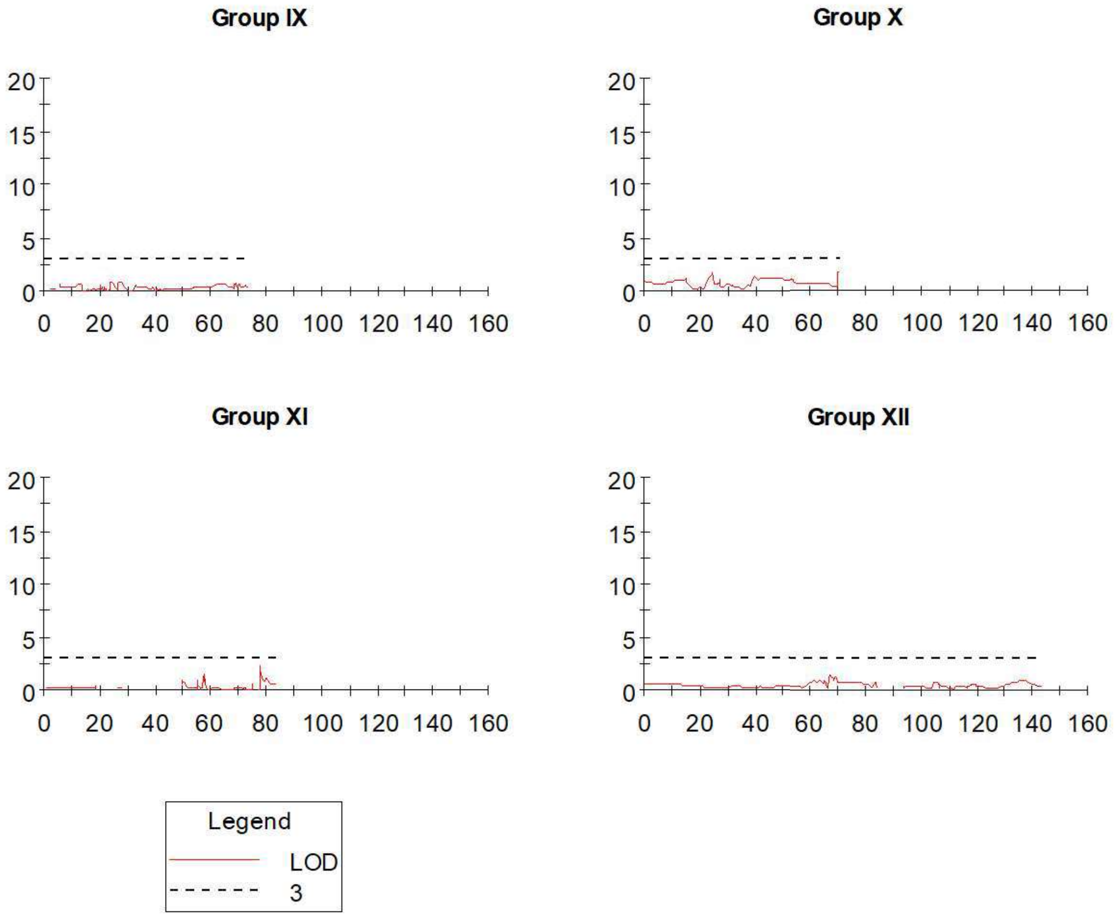

ePGCRURSE5

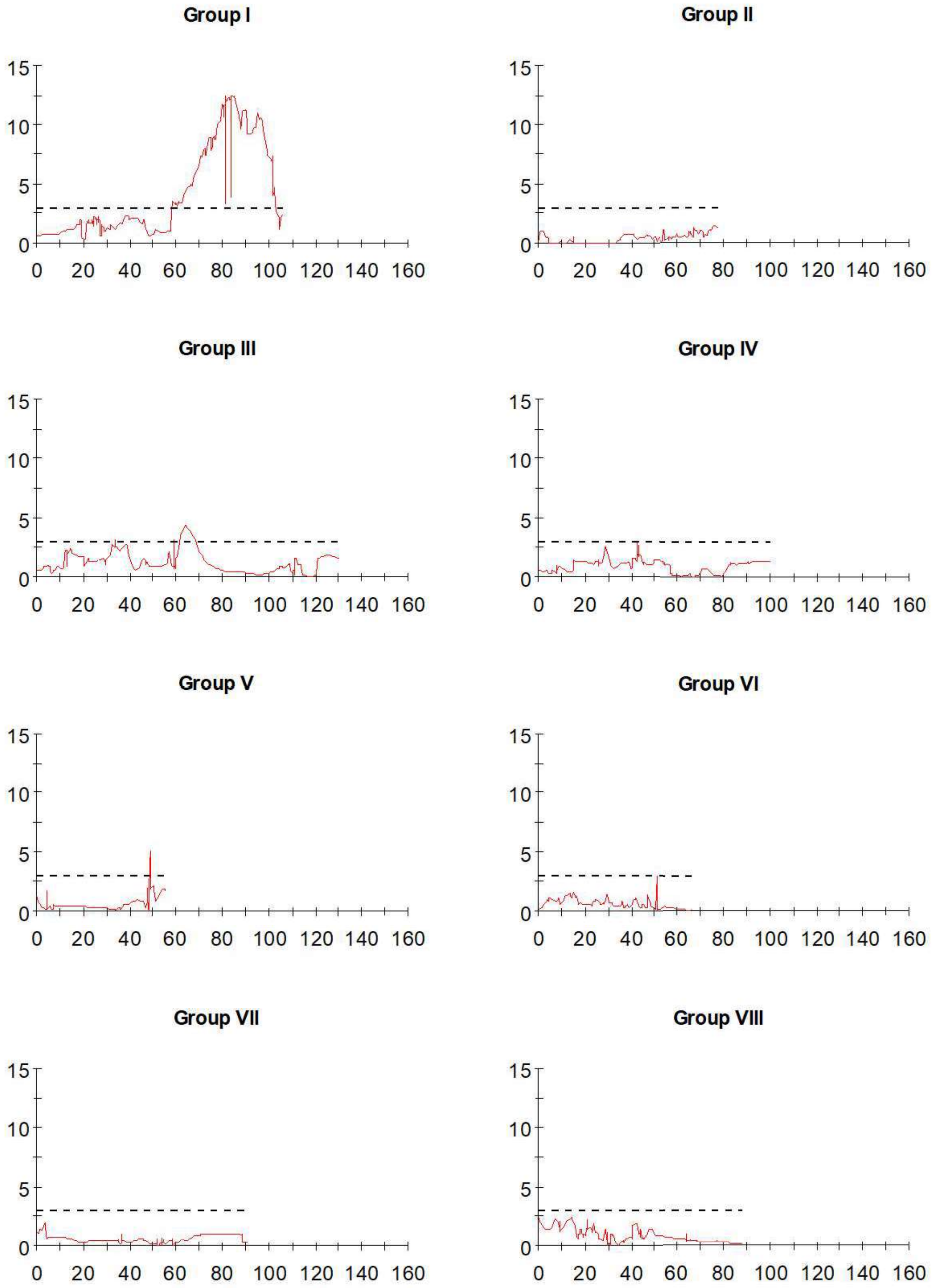

Group I
Group II
Group III
Group IV
Group V
Group VI
Group VII
Group VIII
15
10
5
0
0 20 40 60 80 100 120 140 160

ePGCRURSE5 - continued

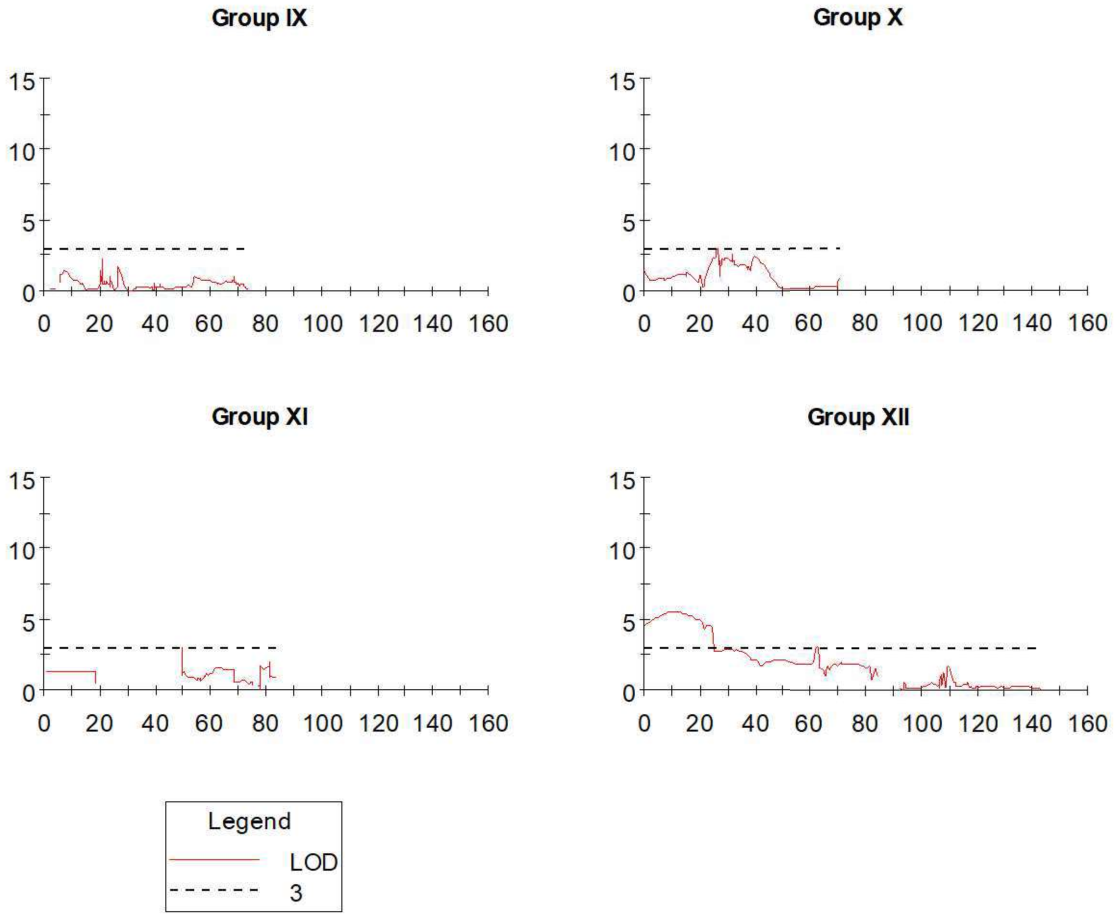

eR1B-23

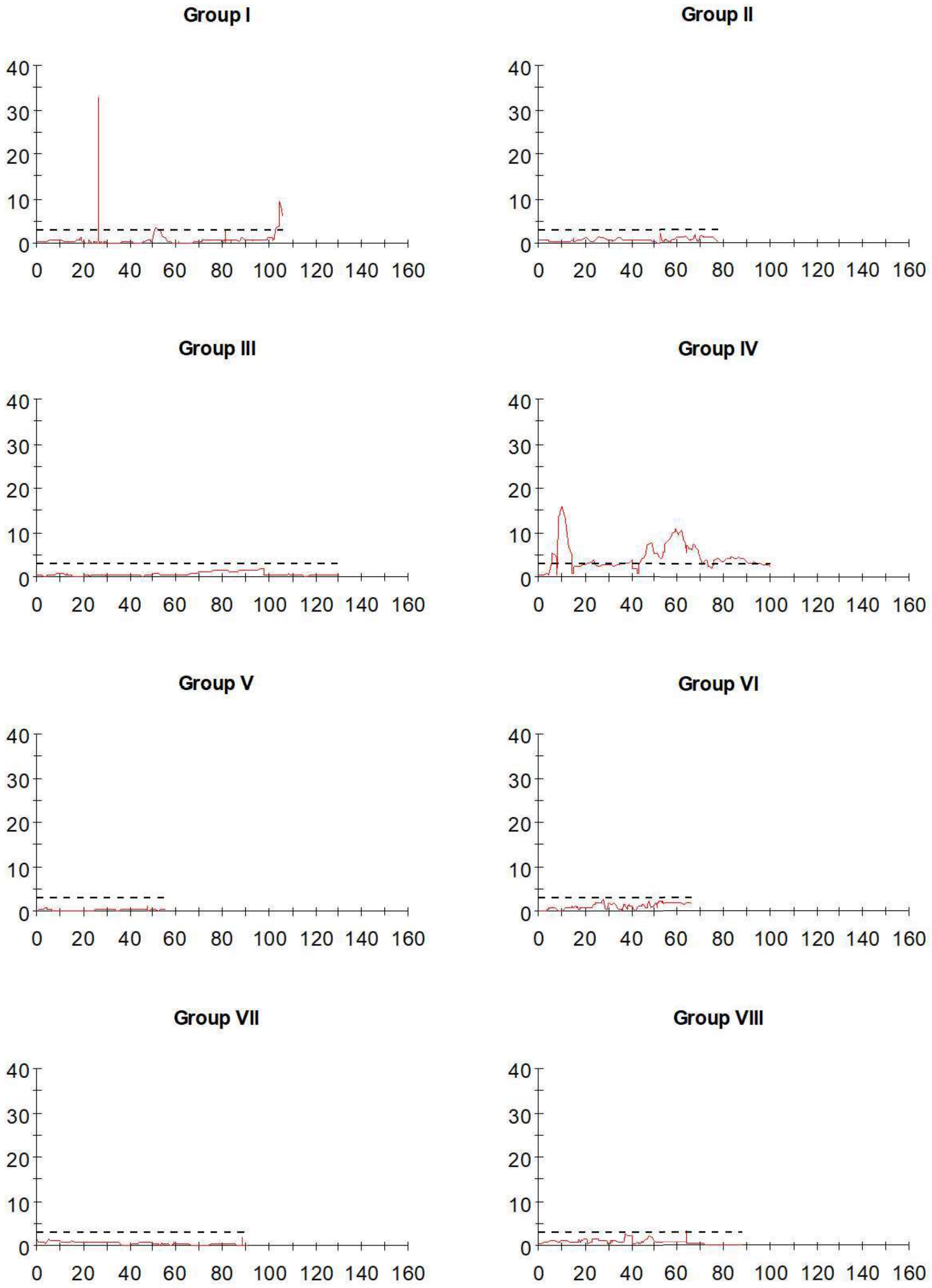
Group I
Group II
Group III
Group IV
Group V
Group VI
Group VII
Group VIII
0
10
20
30
40
0 20 40 60 80 100 120 140 160

eR1B-23 - continued

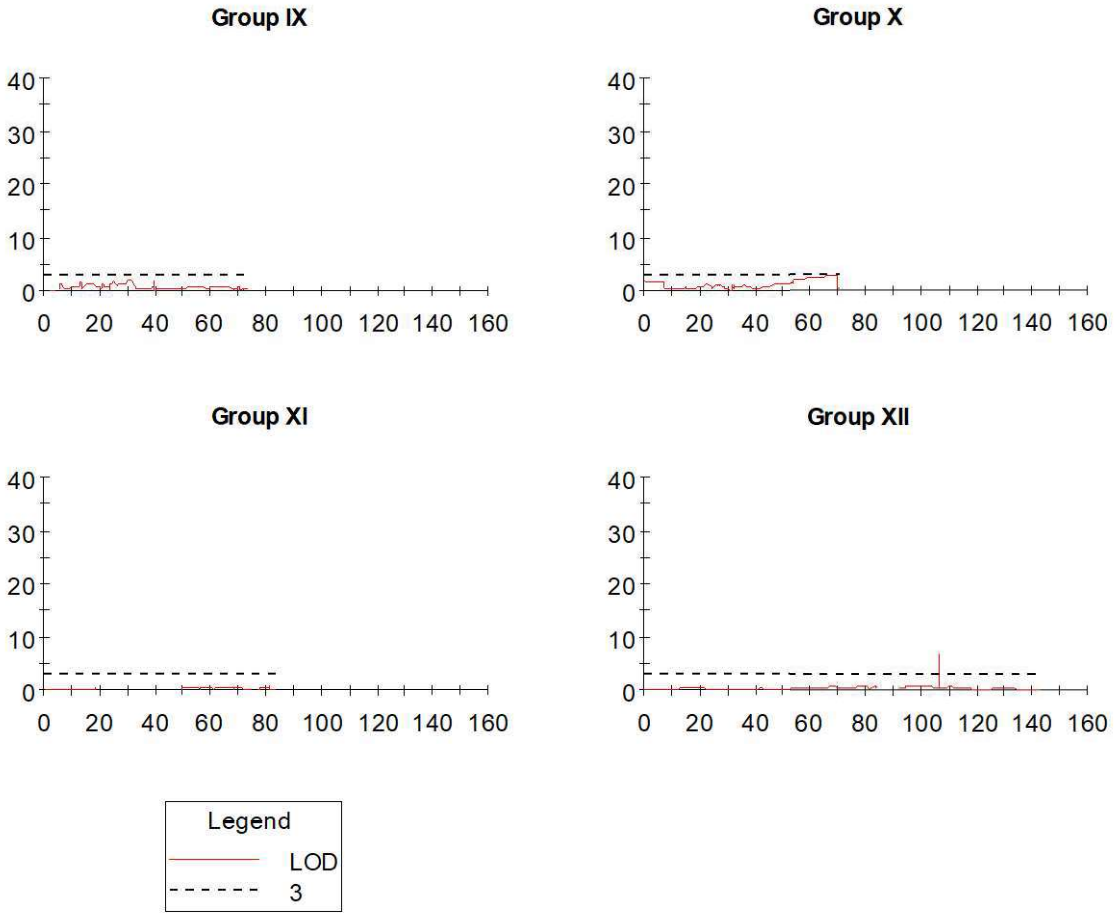

eANR

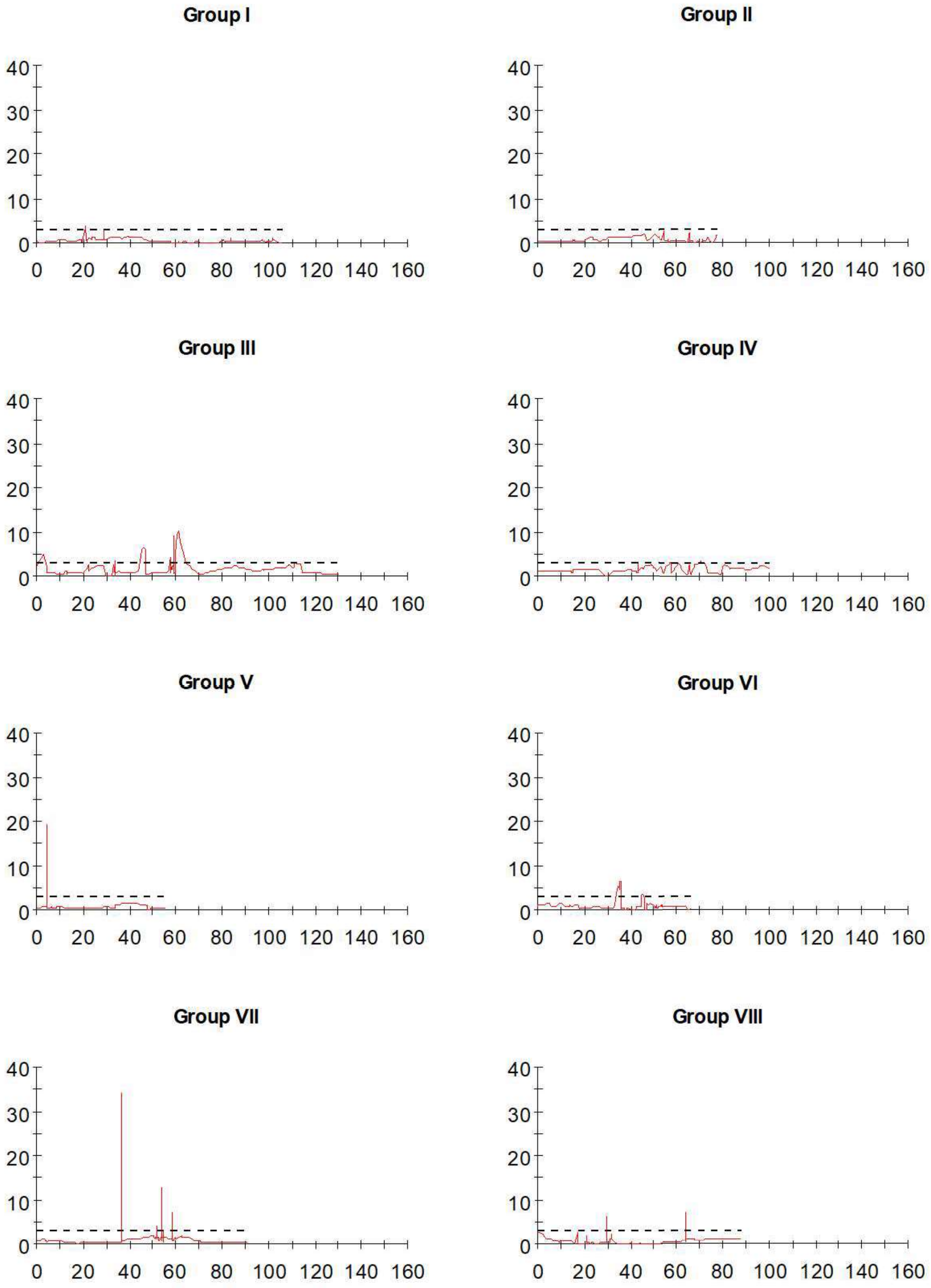
Group I
Group II
Group III
Group IV
Group V
Group VI
Group VII
Group VIII
0
10
20
30
40
0 20 40 60 80 100 120 140 160

eANR - continued

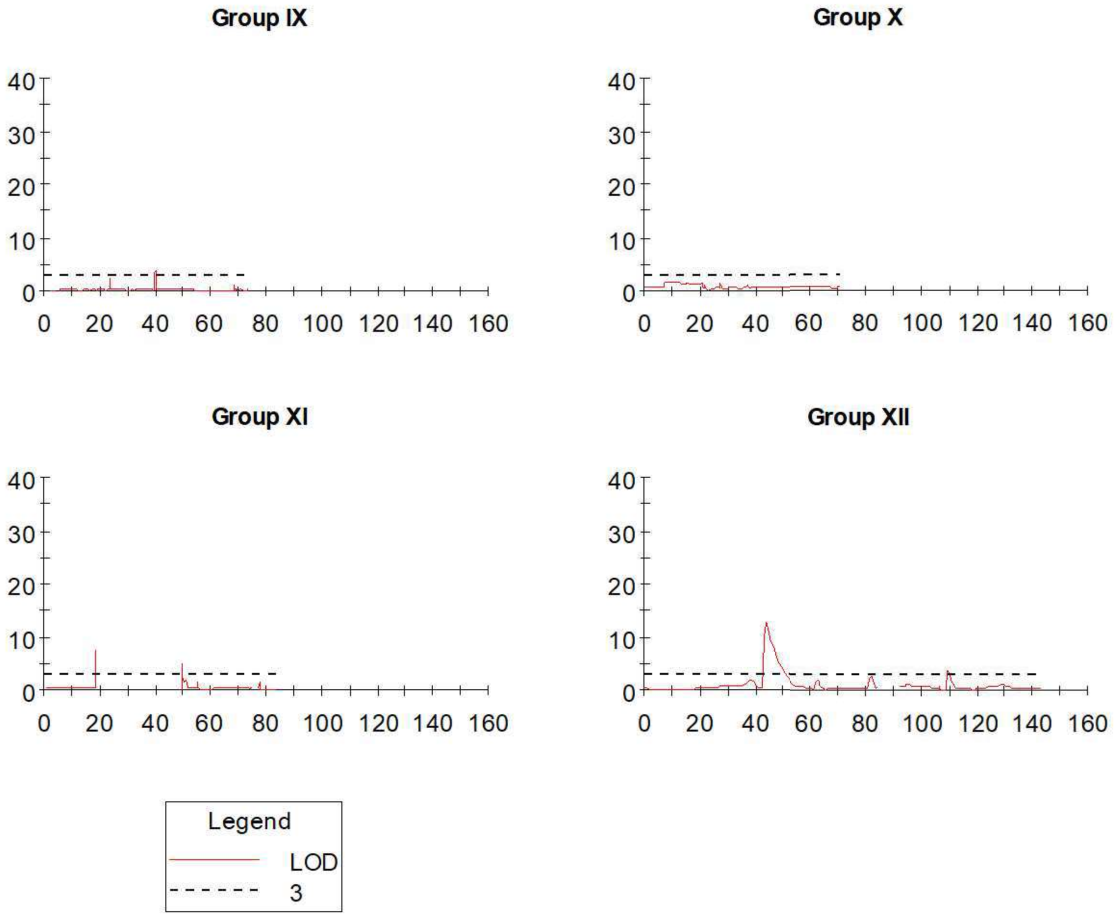

e9-DES

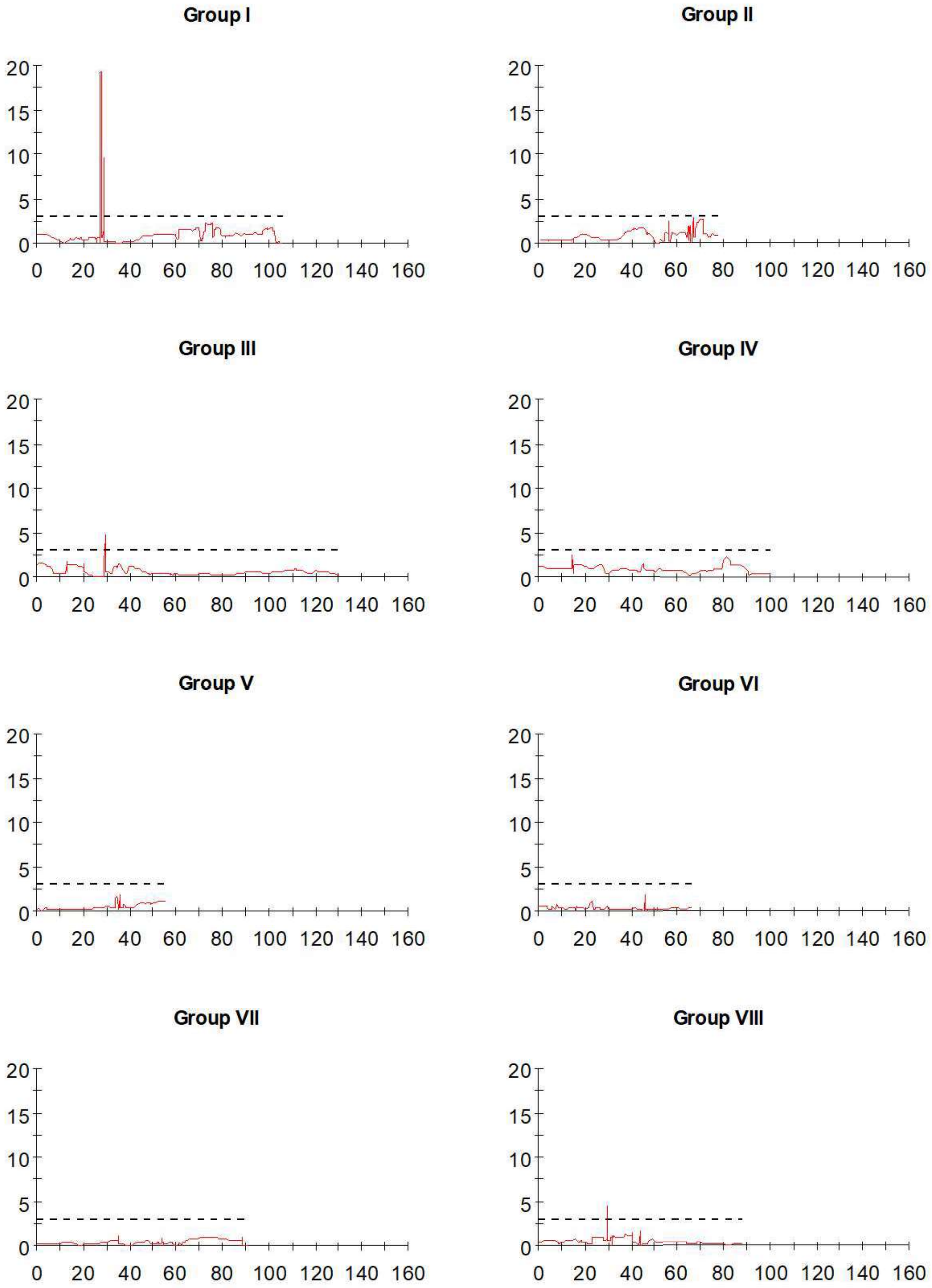
Group I
Group II
Group III
Group IV
Group V
Group VI
Group VII
Group VIII
0
5
10
15
20
0 20 40 60 80 100 120 140 160

e9-DES - continued

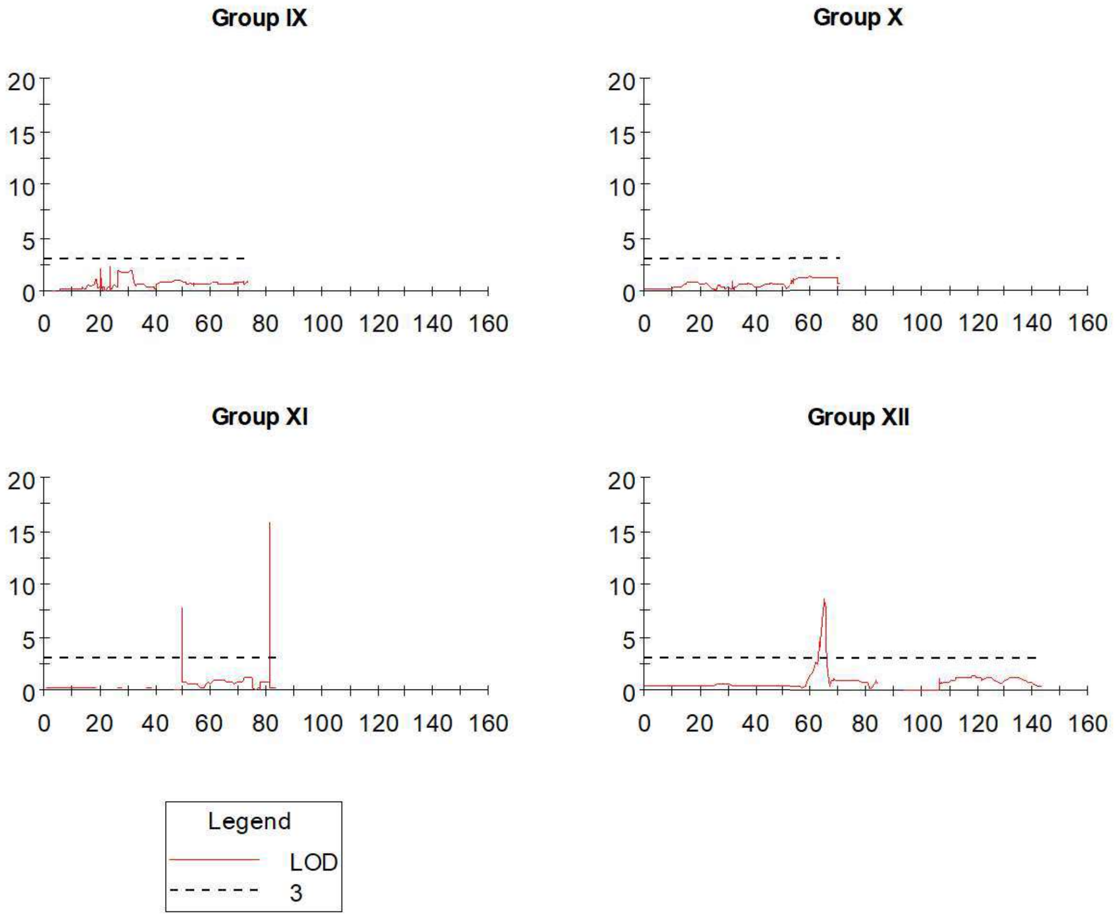

eMLP34

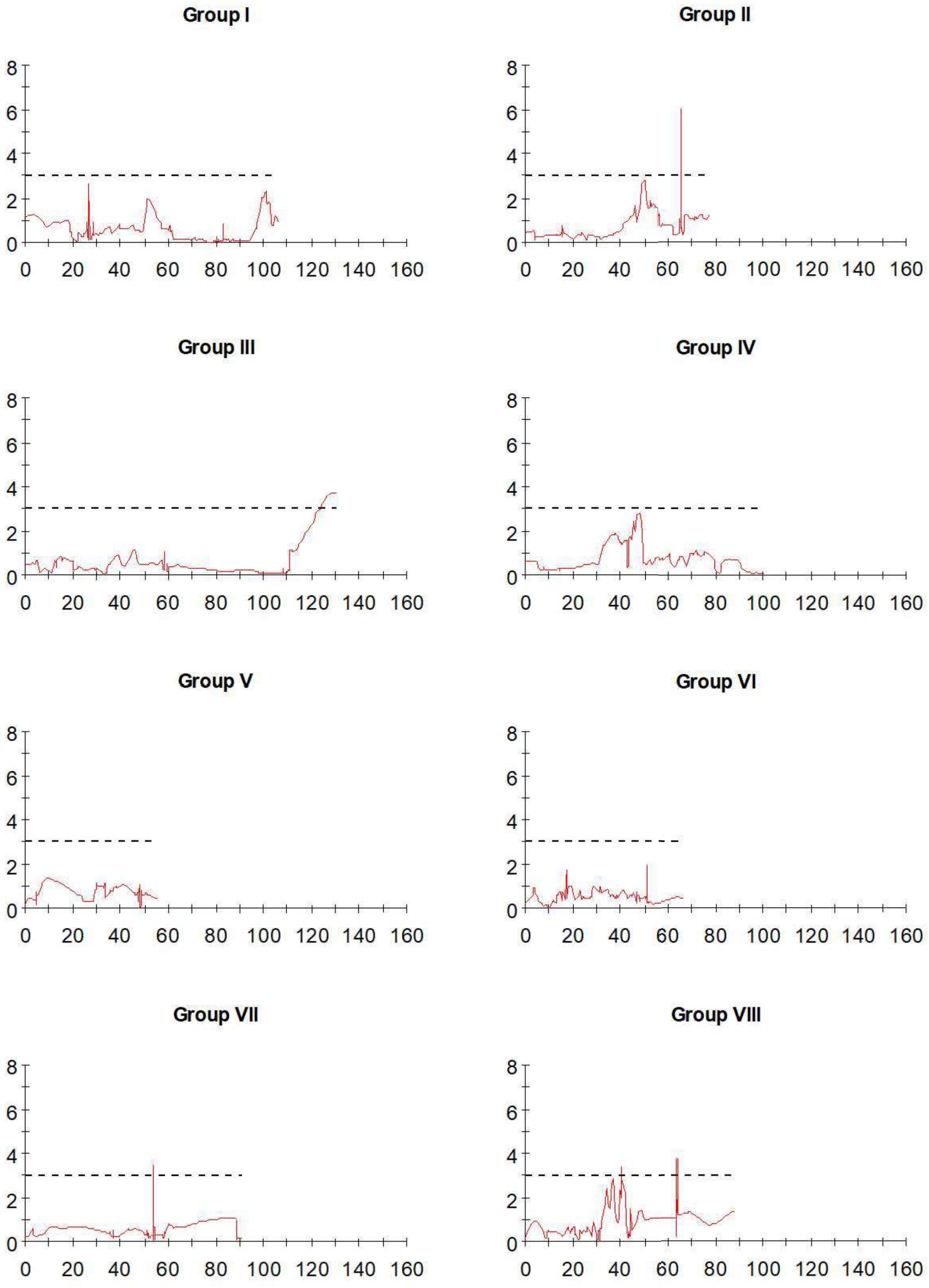
Group I
Group II
Group III
Group IV
Group V
Group VI
Group VII
Group VIII
0
2
4
6
8
0 20 40 60 80 100 120 140 160

eMLP34 - continued

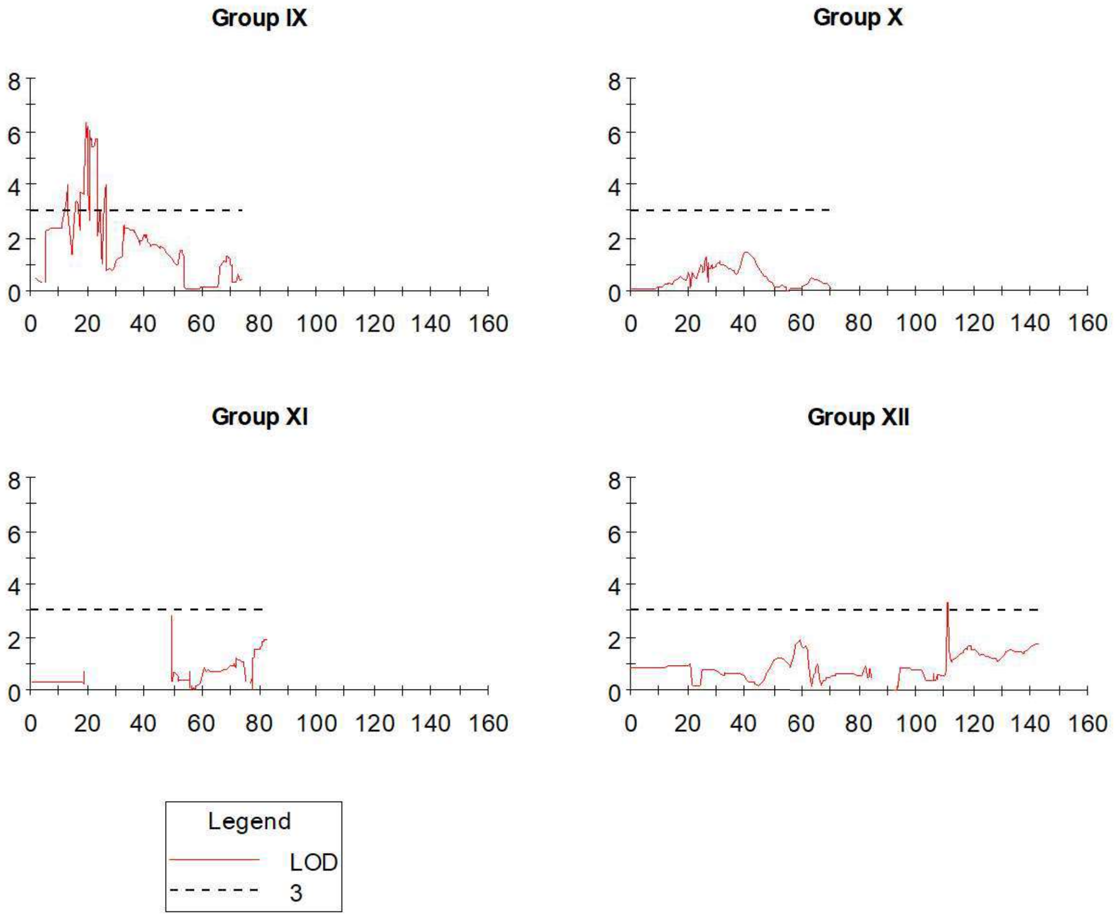

eUnCh835

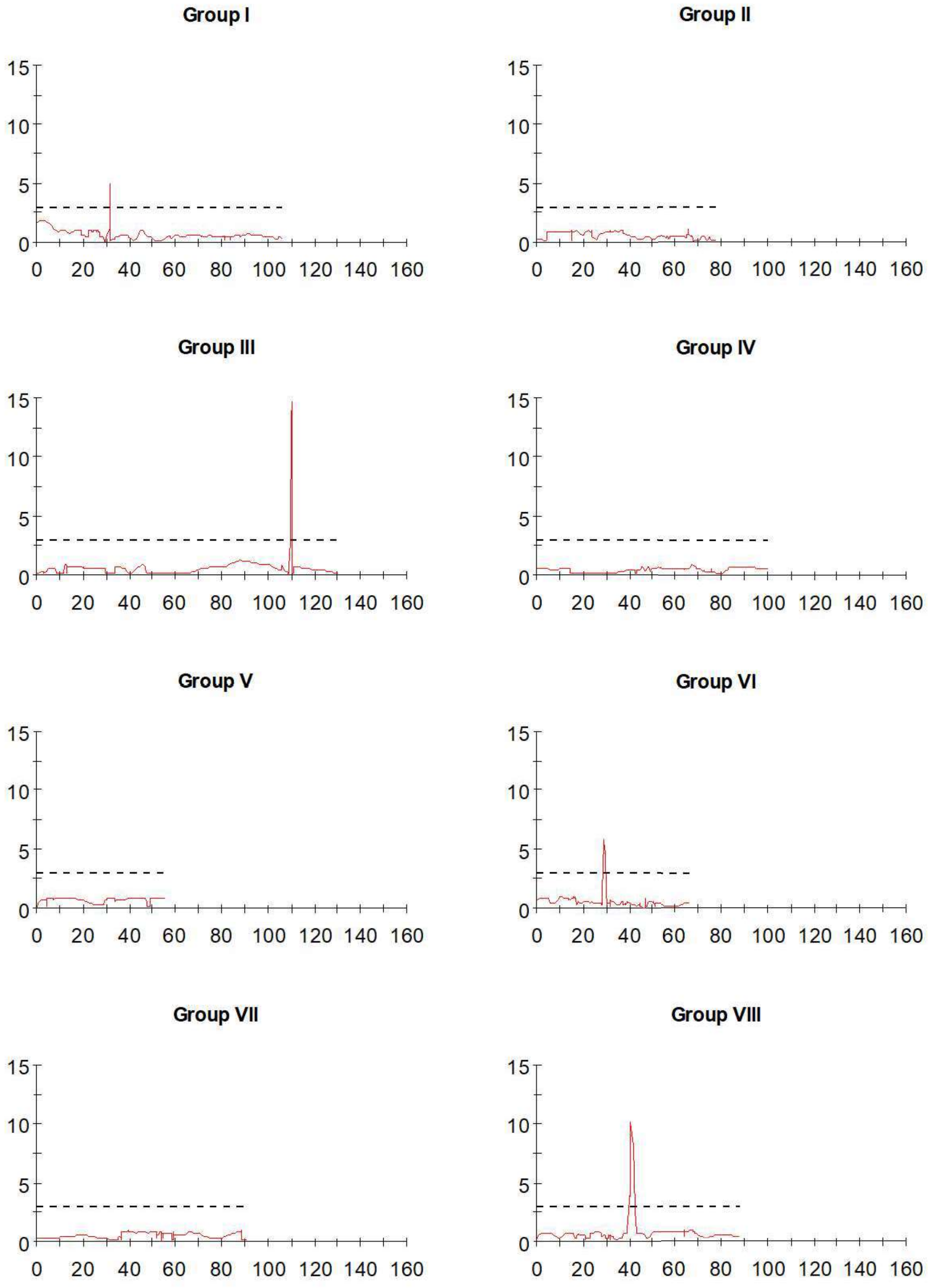

Group I
Group II
Group III
Group IV
Group V
Group VI
Group VII
Group VIII
0
5
10
15
0 20 40 60 80 100 120 140 160

eUnCh835 - continued

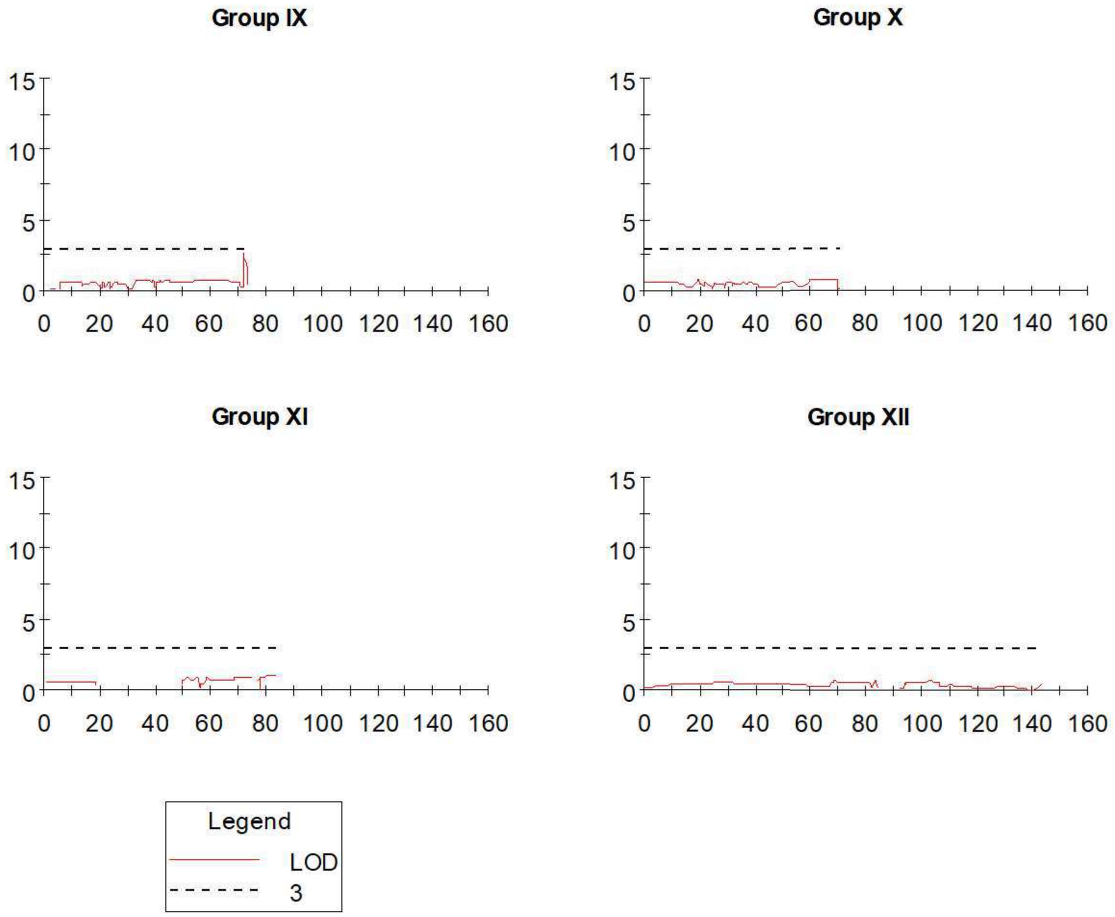

eIRL

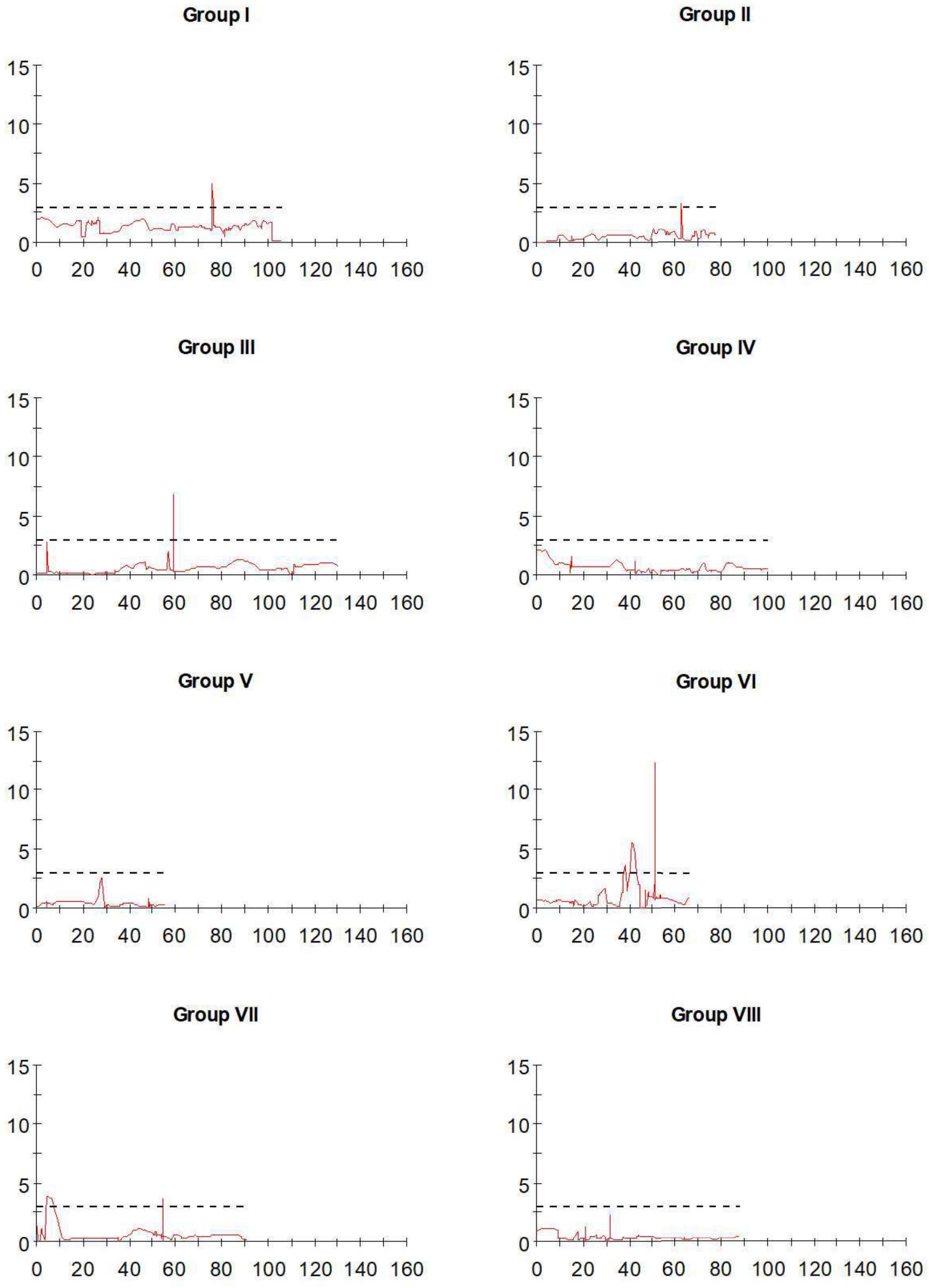
Group I
Group II
Group III
Group IV
Group V
Group VI
Group VII
Group VIII
0
5
10
15
0 20 40 60 80 100 120 140 160

eIRL - continued

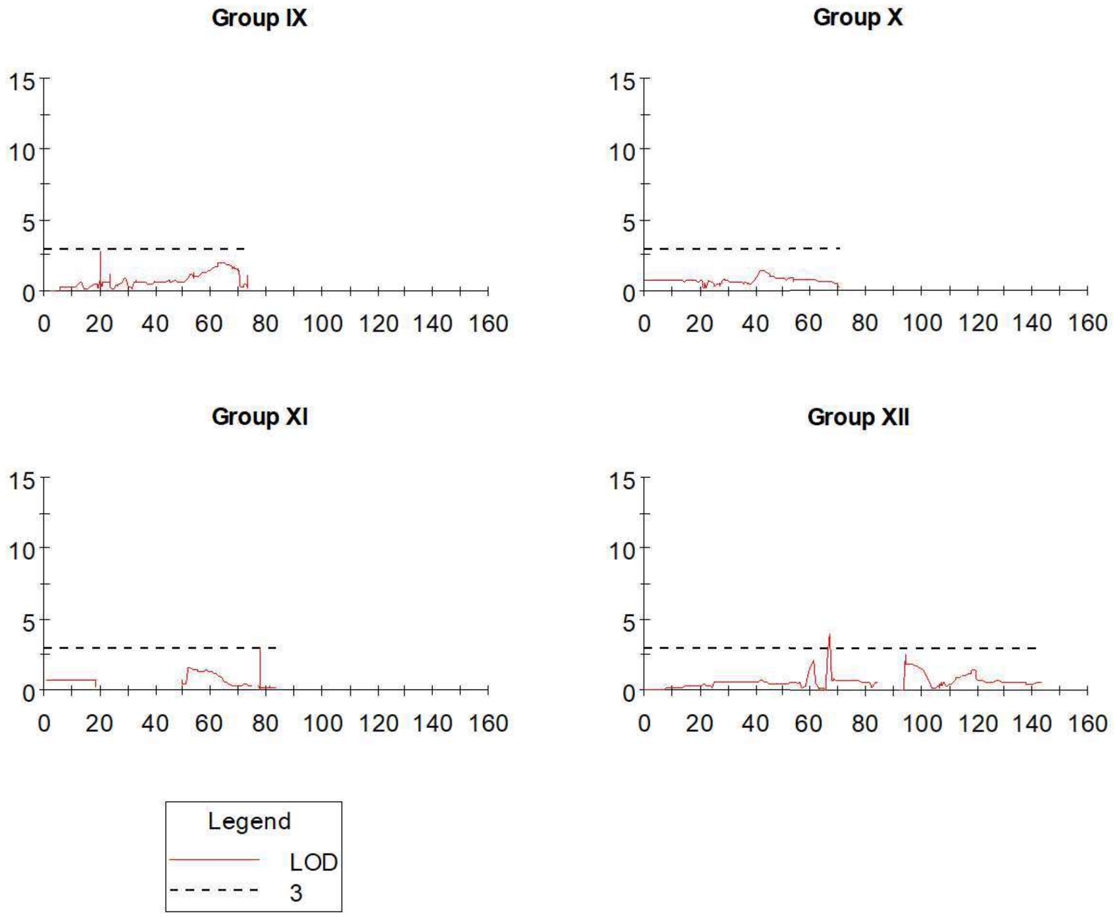

epat3-k1

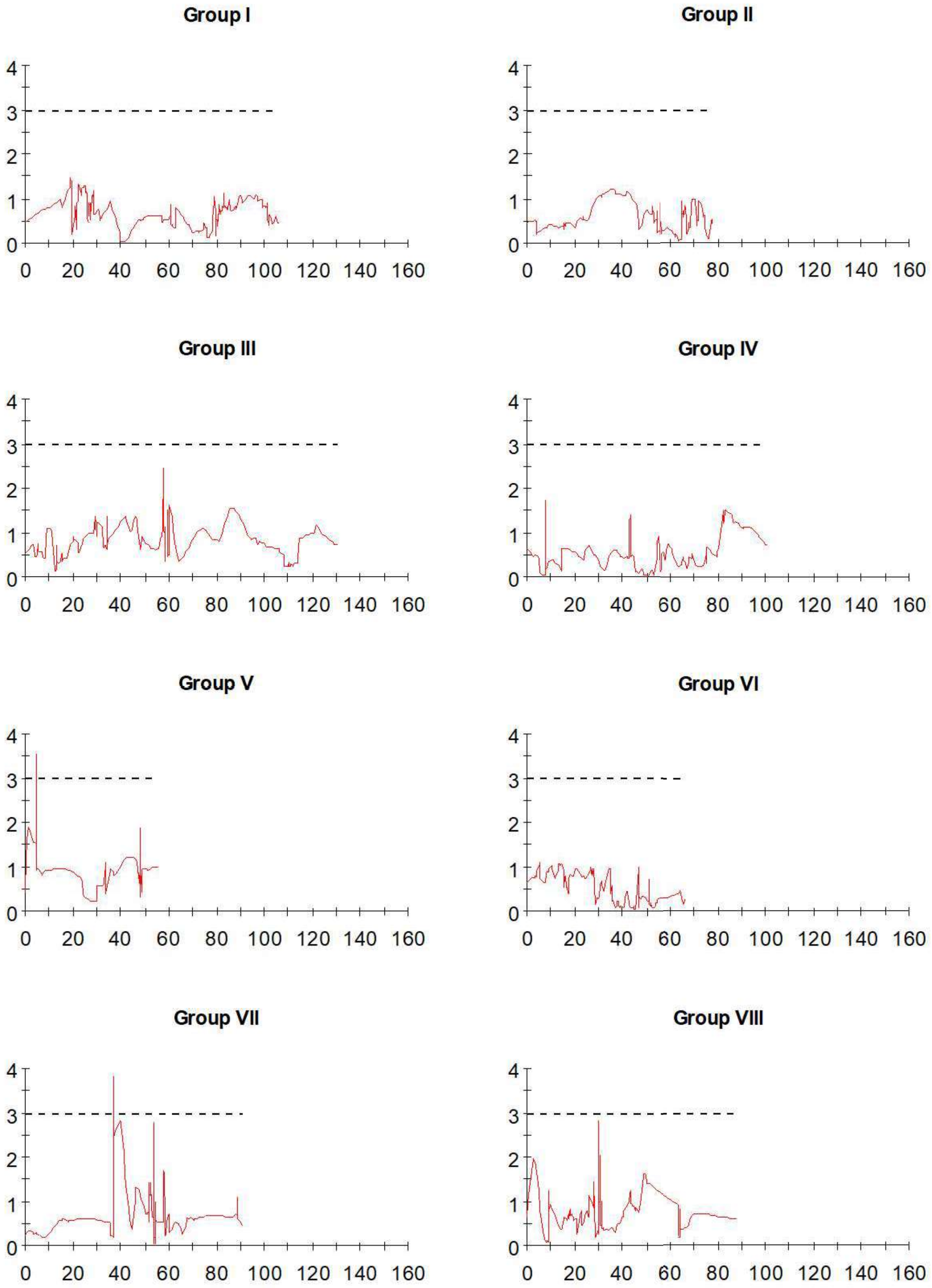
Group I
Group II
Group III
Group IV
Group V
Group VI
Group VII
Group VIII

epat3-k1 - continued

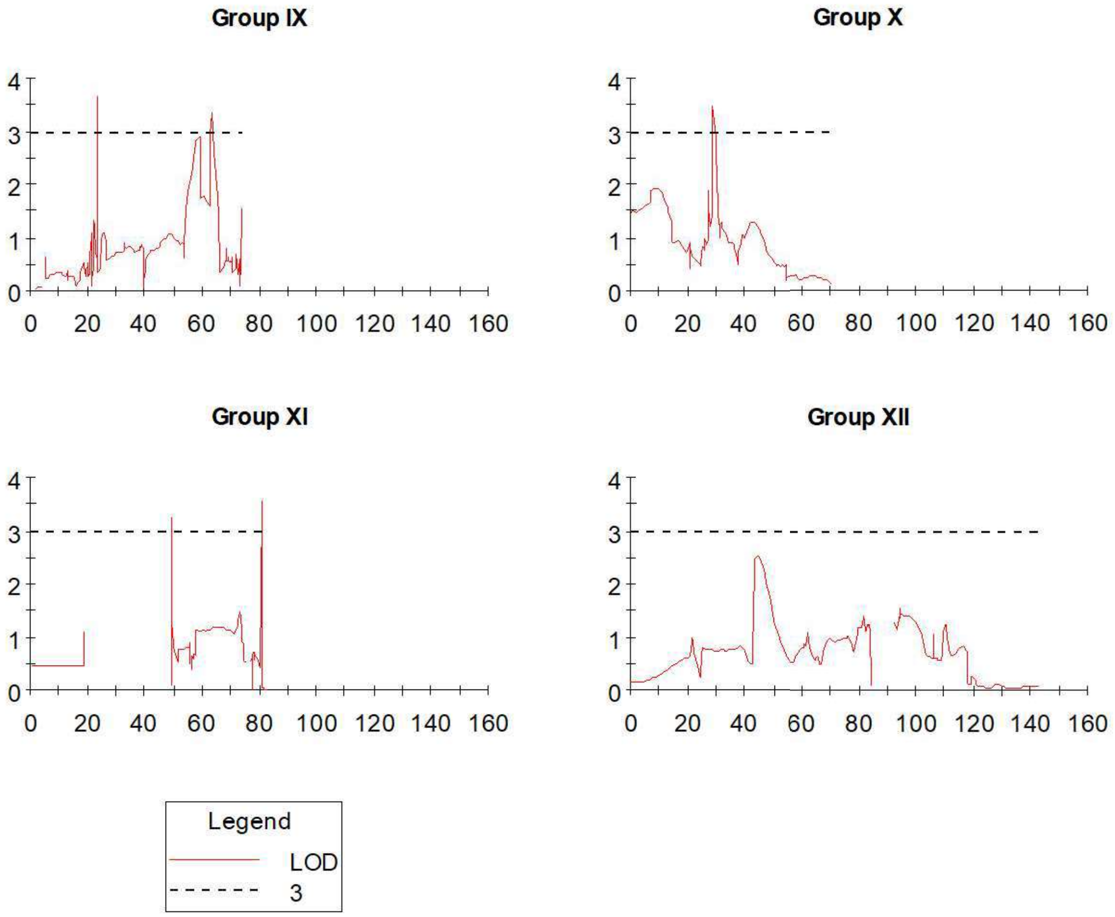

eWAT1

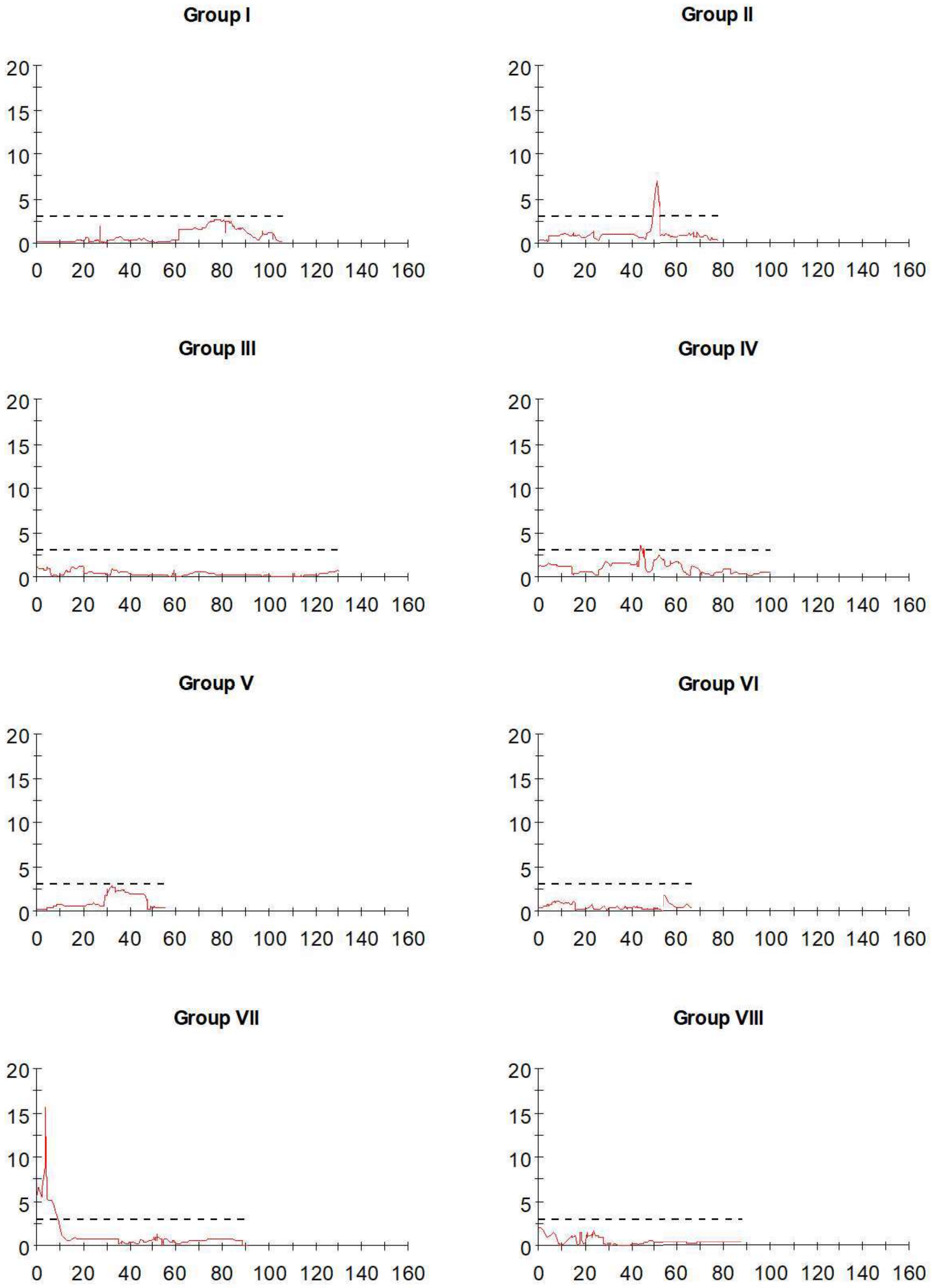
Group I
Group II
Group III
Group IV
Group V
Group VI
Group VII
Group VIII
0
5
10
15
20
0 20 40 60 80 100 120 140 160

eWAT1 - continued

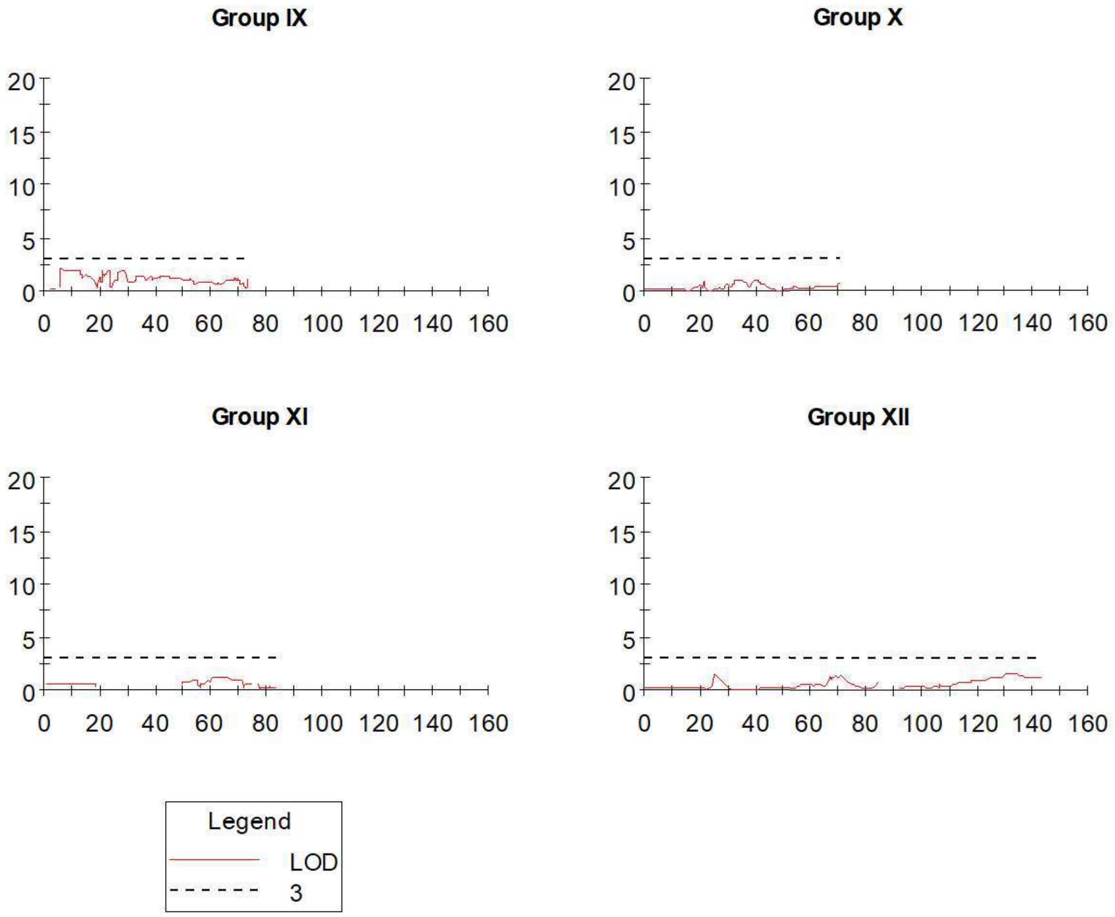

eUnch865

Group I

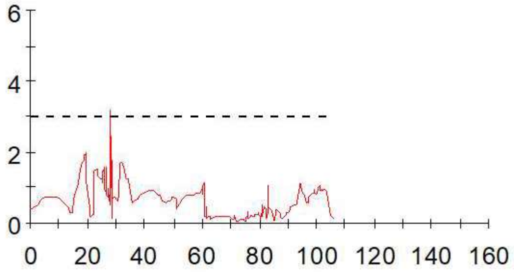

Group II

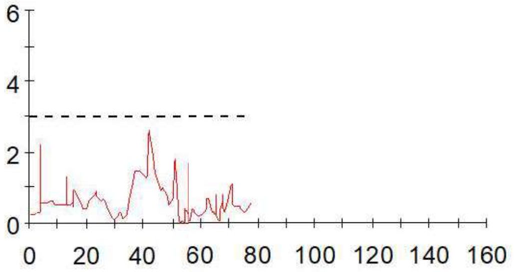

Group III

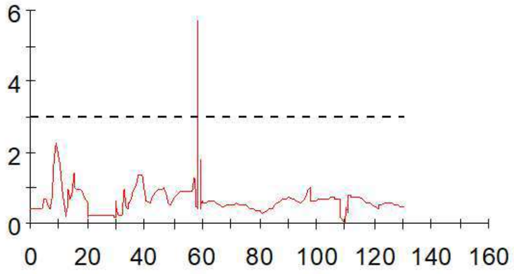

Group IV

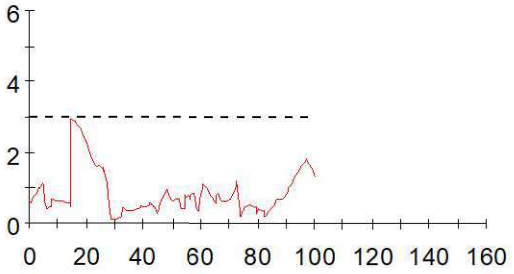

Group V

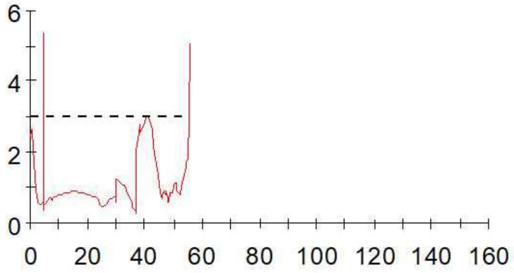

Group VI

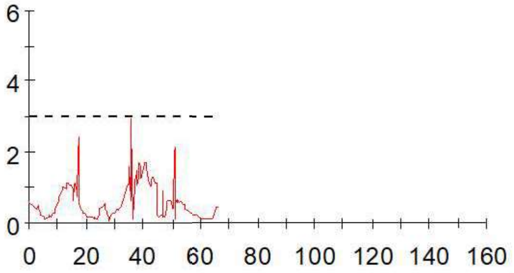

Group VII

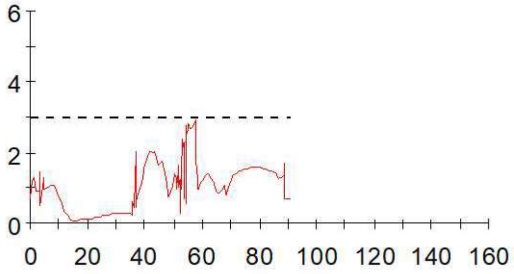

Group VIII

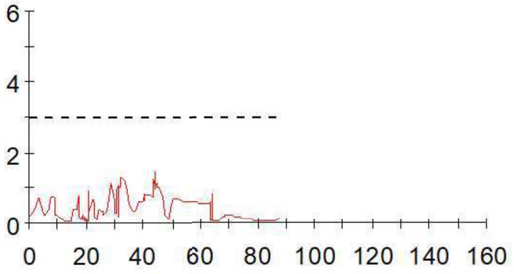

eUnch865 - continued

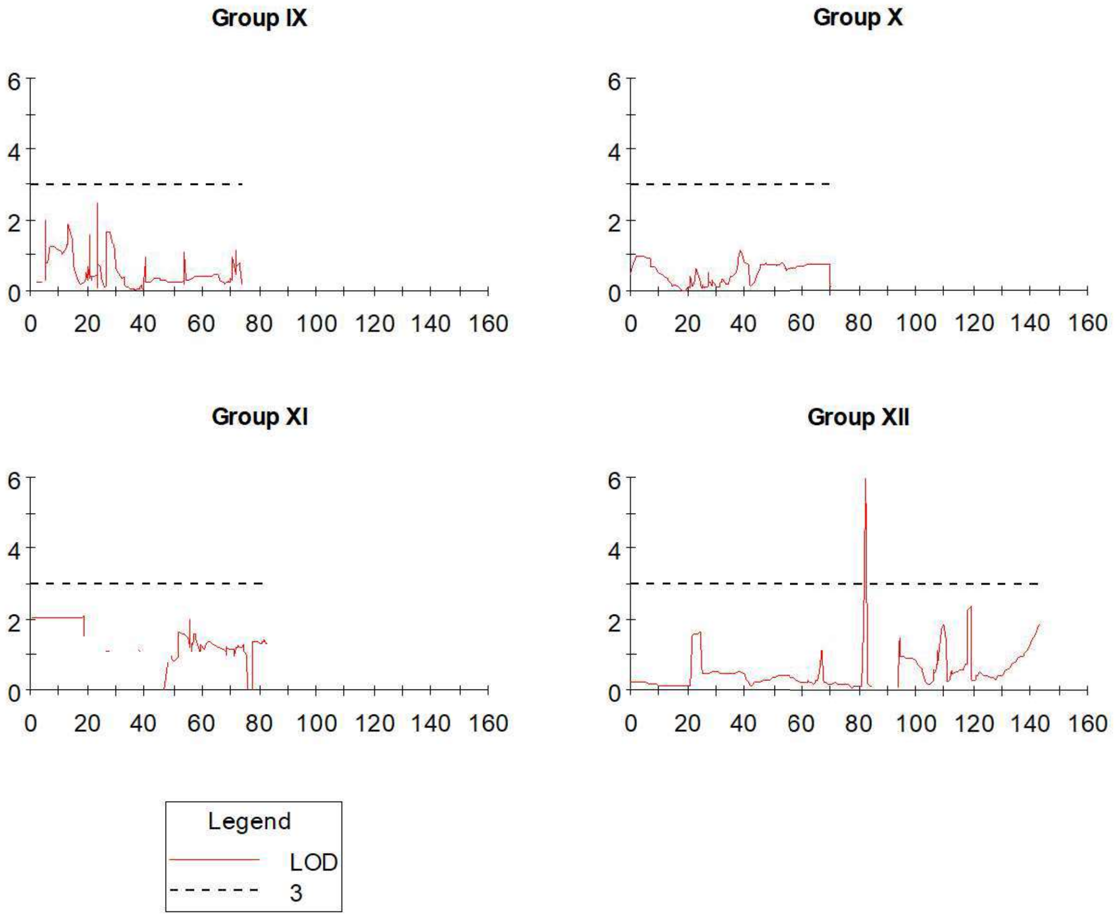

Supplement: Supplementary file 3 — Supplementary Information 3. [file 41598_2020_74285_MOESM3_ESM.pdf]
